# Intergenic splicing-stimulated transcriptional readthrough is suppressed by nonsense-mediated mRNA decay in *Arabidopsis*

**DOI:** 10.1038/s42003-022-04348-y

**Published:** 2022-12-20

**Authors:** Yukio Kurihara, Yuko Makita, Masaharu Kawauchi, Ami Kageyama, Tomoko Kuriyama, Minami Matsui

**Affiliations:** 1 Synthetic Genomics Research Group, RIKEN Center for Sustainable Resource Science, Suehiro-cho 1-7-22, Tsurumi-ku, Yokohama, Kanagawa 230-0045 Japan; 2 Department of Life Sciences, Graduate School of Arts and Sciences, University of Tokyo, Komaba 3-8-1, Meguro-ku, Tokyo 153-8902 Japan; 3 Faculty of Engineering, Maebashi Institute of Technology, Kamisadori 460-1, Maebashi, Gunma 371-0816 Japan; 4 Graduate School of Nanobioscience, Department of Life and Environmental System Science, Yokohama City University, Yokohama, Kanagawa 236-0027 Japan

**Keywords:** RNA quality control, Plant molecular biology

## Abstract

Recent emerging evidence has shown that readthrough transcripts (RTs), including polycistronic mRNAs, are also transcribed in eukaryotes. However, the post-transcriptional regulation for these remains to be elucidated. Here, we identify 271 polycistronic RT-producing loci in *Arabidopsis*. Increased accumulation of RTs is detected in the nonsense-mediated mRNA decay (NMD)-deficient mutants compared with wild type, and the second open reading frames (ORFs) of bicistronic mRNAs are rarely translated in contrast to the first ORFs. Intergenic splicing (IS) events which occur between first and second genes are seen in 158 RTs. Splicing inhibition assays suggest that IS eliminates the chance of transcription termination at the polyadenylation sites of the first gene and promotes accumulation of RTs. These results indicate that RTs arise from genes whose transcription termination is relatively weak or attenuated by IS, but NMD selectively degrades them. Ultimately, this report presents a eukaryotic strategy for RNA metabolism.

## Abstract (web summary)

Readthrough transcripts (RTs) are identified in the genome of *Arabidopsis* using a long-read sequencing technology and structural and fate examination reveals that RTs are post-transcriptionally regulated by splicing, transcription termination and NMD.

## Introduction

Post-transcriptional regulatory mechanisms are important determinants for the fate of transcripts^1^. Of these mechanisms, nonsense-mediated mRNA decay (NMD) is a translation-dependent mRNA quality control mechanism that selectively eliminates some kinds of aberrant mRNAs in eukaryotes including plants^2^. From our present knowledge, it is known that some features, such as upstream open reading frames (ORFs), premature termination codons, relatively long 3′ untranslated regions (3′ UTRs), and short ORFs on a long noncoding RNA, can be triggers for NMD^3–6^. In addition, it is well-known that NMD is associated with splicing because alternative splicing sometimes produces an incorrect transcript that harbors a premature termination codon^7^. Up-frameshift proteins, UPF1, UPF2, and UPF3, are essential factors for NMD and play a role in detecting the features of NMD targets^2^. Disruption of NMD in *Arabidopsis thaliana* causes morphological defects or, in some cases, embryonic lethality^8–10^, probably because unexpected accumulation of the targets negatively interferes with various vital activities and disturbs homeostasis. In fact, previous transcriptomic work has revealed over-accumulation of several kinds of NMD targets in *upf1*, *upf2*, *upf3*, and *smg7* knockdown mutants^3,7,11,12^. However, the specificity of NMD targets still remains unclear.

Generally, eukaryotic transcriptional units produce monocistronic mRNAs, whereas polycistronic mRNAs, which possess multiple ORFs, are commonly seen as operons in prokaryotes and the prokaryote-originating organelles, mitochondria, and chloroplasts^13^. Recent emerging evidence derived from long-read sequencing technologies has shown that not only monocistronic mRNAs and their isoforms but also many readthrough transcripts (RTs), including polycistronic mRNAs, are present in some eukaryotes^14–19^. Proteomic analysis has indicated that the second ORFs as well as the first ORFs in polycistronic mRNAs are also translated in green algae^20^. Another report in *Arabidopsis* showed that both genes co-transcribed into a bicistronic mRNA play their independent roles; *CDC26*, a first gene, regulates the cell cycle and *TTM3*, a second gene, assists in *CDC26* translation^21^. However, elucidation of their post-transcriptional regulation and biological roles is just getting started.

Transcription termination of RNA polymerase II-transcribed genes and polyadenylation of their mRNAs are properly regulated. Polyadenylation signals are typically located around +50 nt relative to the polyadenylation site in eukaryotes. Transcribing pre-mRNA undergoes endonucleolytic cleavage and polyadenylation at the cleavage site. Subsequently, transcription is terminated by XRN3 exonuclease-mediated 5′-3′ degradation of a downstream readthrough remnant in plants^22–24^. Although AAUAAA is the most frequently used polyadenylation signal in mammals, its usage in plants is much lower (about 10% in *Arabidopsis*)^25^. In contrast, plant polyadenylation requires the cooperative effort of various 3′UTR elements, such as far-upstream elements (FUEs), near-upstream elements (NUEs), cleavage elements (CEs), and U-rich regions, which are distributed in +200 nt relative to the polyadenylation site^25–27^.

Here, we identified RTs throughout the genomes of young seedlings of wild-type *Arabidopsis* and *upf1-1*, an NMD-deficient mutant, using a long-read sequencing technology and analyzed their biogenesis and metabolism. Our analysis suggests that RTs are post-transcriptionally regulated by splicing, transcription termination, and NMD.

## Results

### Identification of readthrough transcripts in wild-type *Arabidopsis* and *upf1-1*

Isoform sequencing (ISO-seq), long-read sequencing analysis using a PacBio Sequel II platform, was performed for mRNAs derived from 3-day-old *Arabidopsis* wild-type (WT) and *upf1-1* seedlings, that were either grown in the dark or blue-light irradiated following growth in darkness. The analysis identified a total of 271 loci that produced readthrough transcripts (RTs) overlapping two or more protein-coding genes (Table 1 and Supplementary Data 1). Of them, RTs in 217 loci were also detected in the same RNA samples by Nanopore long-read sequencing (Supplementary Data 1). In addition, RTs in 157 loci had already been detected in previous work using Nanopore sequencing in 14-day-old wild-type seedlings^17^, indicating that these RTs are not limited to early developmental stages. The loci are uniformly distributed throughout the five chromosomes except in heterochromatic regions (Supplementary Fig. 1a). In *upf1-1*, RTs were detected two or more times more frequently than those in WT (Table 1) and 165 of the 271 loci were seen only in *upf1-1* (Supplementary Fig. 1b), suggesting the possibility that RTs are a kind of NMD target.

**Table 1:** Summary of ISO-seq analysis for identification of RTs.

Sample	Number of RT variants	Number of RTs	Number of RT loci	Total number of transcripts identified in ISO-seq	Proportion of RTs to total transcripts (%)
WT_Dark	65	185	47	290648	0.064
WT_Blue	108	300	83	478433	0.063
upf1_Dark	391	1413	205	517589	0.273
upf1_Blue	225	723	150	424341	0.170

**Fig. 1:**
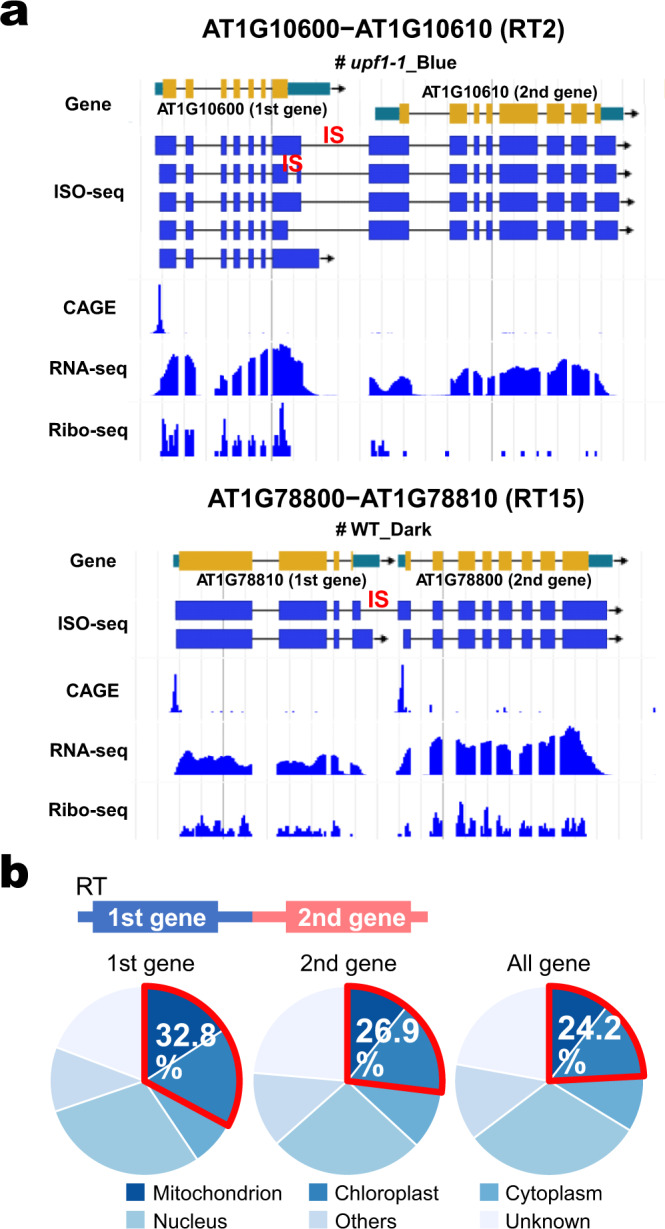
Identification of readthrough transcripts (RTs) in young *Arabidopsis* seedlings by long-read sequencing. **a** Example display of two RT loci (AT1G10600-AT1G10610 (RT2) and AT1G78800-AT1G78810 (RT15)). IS = intergenic splicing. **b** Localization prediction of products of the first and second genes of the 271 identified RT loci and all genes.

In 195 and 158 of the 271 RT loci, monocistronic transcripts independently derived from first and second genes, respectively, were also detected in the ISO-seq analysis. From CAGE (capped analysis of gene expression) analysis^28^, transcription start sites (TPM > 0.2) for 194 second genes were detected and, indeed, earlier CAGE analysis in 10-day-old seedlings^4^ also detected transcription start sites for 183 second genes. In summary, transcription start sites for 202 second genes were detected in either or both growth stages (Supplementary Data 1). These observations confirmed separate monocistronic transcriptions in addition to polycistronic transcriptions under our growth conditions. Curiously, intergenic splicing (IS) events, which occur between the 3′ region of the first gene and the 5′ region of the second gene, were detected in 158 RT loci, exemplified in Fig. 1a.

Whether or not transcription termination of the first gene occurs is responsible for readthrough transcriptional elongation. Prediction analysis of products of the first and second genes reveal that localization at mitochondria or chloroplasts is relatively enriched in first genes of the RT loci (89/271) compared with distribution of the second (73/271) and all genes (Fig. 1b). The hypergeometric test for the enrichment of the 89 first genes encoding mitochondria or chloroplast-localized proteins represented a *p*-value < 0.001, which is sufficient to guarantee the enrichment. Of these, 28 first genes encode pentatricopeptide repeat proteins (PPRs) or tetratricopeptide repeat proteins (TPRs). This result suggests that transcriptional readthrough events likely occur in prokaryote-originating genes. Also, in 28 RT loci, mitochondrion- or chloroplast-localized proteins are encoded in both first and second genes.

### RTs are over-accumulated in *upf* NMD-deficient mutants

**Fig. 2:**
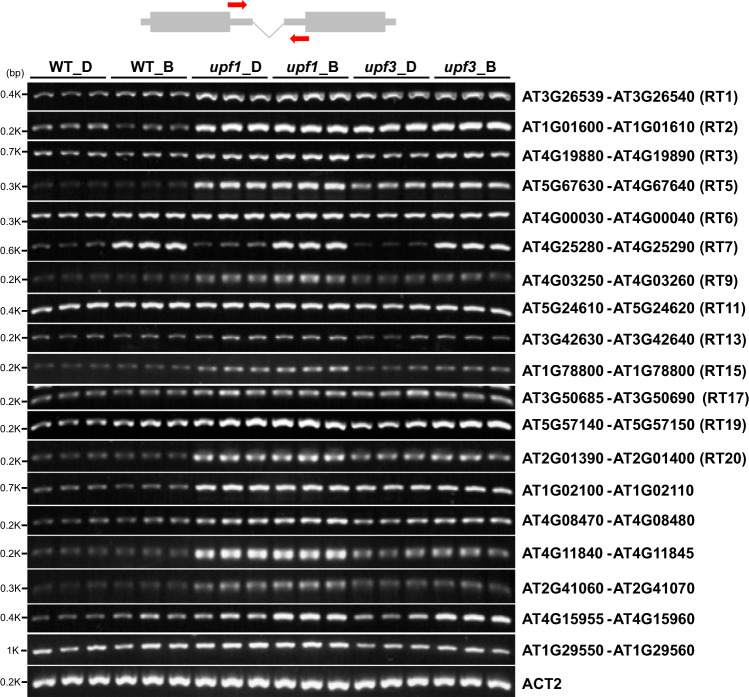
Accumulation of bicistronic RTs in the NMD-deficient mutants, *upf1-1* and *upf3-1*. Results of semi-quantitative RT-PCR for 19 selected bicistronic RTs using primers (upper panel) that amplify the joint region between the first and second genes are shown. *ACT2* was used as an internal control. *n* = 3 biologically independent samples. D = Dark, B = Blue.

The higher detection rate of RTs in *upf1-1* (Table 1) suggested that most of the RTs may be targeted by NMD. To validate this possibility, accumulation of 19 selected polycistronic RTs (listed in Supplementary Data 1) was compared between wild type (WT), *upf1-1* and *upf3-1*. Semi-quantitative reverse transcription polymerase chain reaction (RT-PCR) using primers designed to amplify the joint regions between the first and second genes was used in this study, which can distinguish and separately detect pre-RTs with IS introns and RTs without them. The RT-PCR results, as expected, showed that most of the RTs examined were more accumulated in *upf1-1* and/or *upf3-1* than in WT (Fig. 2). This result confirmed the conviction that RTs are targets of NMD.

Note that 13 (RT1, RT2, RT3, RT5, RT6, RT7, RT9, RT11, RT13, RT15, RT17, RT19, and RT20) of the selected bicistronic RTs were used for following analysis.

### The termination codon of a first ORF is associated with NMD

NMD occurrence is tightly associated with translational state^2^. To investigate the translation of the first and second ORFs on polycistronic RTs, ribosome profiling analysis (Ribo-seq), where ribosome footprints are sequenced^29,30^, was applied to dark-grown and blue light-irradiated WT and *upf1-1*. Unfortunately, it was impossible to distinguish translation of bicistronic RTs from that of monocistronic mRNAs independently derived from each gene as exemplified in Fig. 1a (Supplementary Fig. 2).

**Fig. 3:**
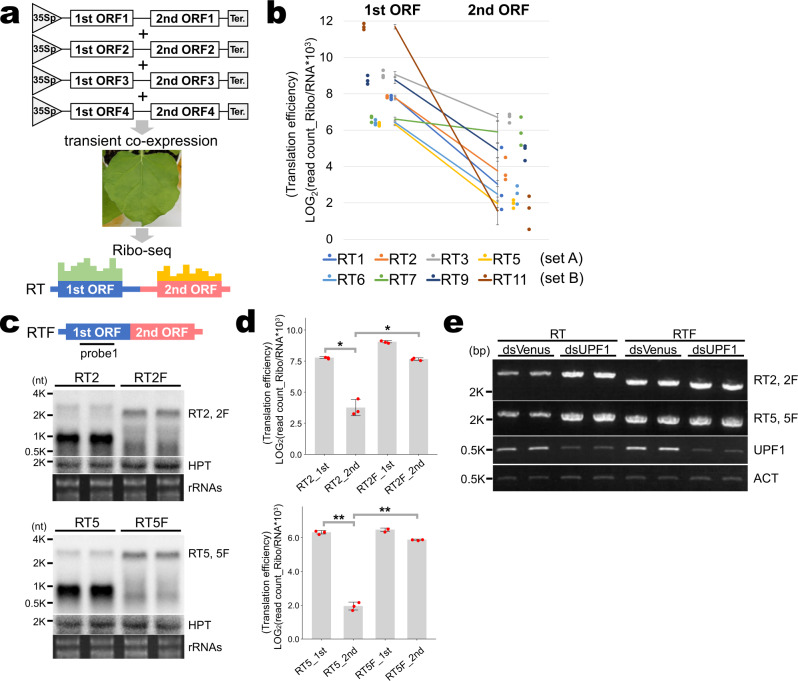
Survey for the relationship between translation and NMD. **a** Methodology for transient expression assay. Four RTs were transiently expressed at the same time and analyzed by Ribo-seq. **b** Comparison of translation efficiency between the first and second open reading frames (ORFs). Set A (RT1, RT2, RT3, and RT5) and Set B (RT6, RT7, RT9, and RT11) were tested separately. *n* = 3 biologically independent samples. Individual data points are shown on the left (first ORF) and right (second ORF) sides of the graph. Error bars show the standard deviation. **c** Northern blot analysis for the detection of RT2, RT2F, RT5, and RT5F. Total RNAs were extracted from *Nicotiana benthamiana* leaves at 3 days after infiltration. *HPT* (hygromycin phosphotransferase) was also detected as a loading control. The *HPT* gene was on the same expression vector as the RTs and RTFs. *n* = 2 biologically independent samples. **d** Translation efficiency of the first (1st) and second (2nd) ORFs of RT2 and RT2F (upper panel), and RT5 and RT5F (lower panel). *n* = 3 biologically independent samples. Asterisks indicate significant differences (Tukey’s test; *P < 10^−5^, **P < 10^−6^). Individual data points are shown as red dots. Error bars show the standard deviation. **e** RT-PCR analysis for the detection of RT2, RT2F, RT5 and RT5F in a transient RNAi assay. RTs and RTFs were co-expressed with either dsUPF1 or dsVenus. Total RNA was extracted from *Nicotiana benthamiana* leaves at 6 days after infiltration. *NbACT* expression was used as a loading control. *n* = 2 biologically independent samples.

To overcome this difficulty and to learn the steady-state translation of the first and second genes, four RTs were transiently co-expressed in leaves of *Nicotiana benthamiana* by agroinfiltration and their levels of translation were measured by Ribo-seq (Fig. 3a). Eight RTs (RT1, RT2, RT3, RT5, RT6, RT7, RT9, RT11) were used for the assay, divided into two sets. In the transient assay, accumulation of full-length RTs for a few RTs like RT3 and RT7 were only faintly detected, and accumulation of shorter transcripts were detected for some RTs (Supplementary Fig. 3). We could not rule out the possibility that the shorter transcripts (Supplementary Fig. 3) are first monocistronic mRNAs resulted from unexpected transcription termination. Therefore, to appropriately evaluate translation state of the first and second ORFs, translation efficiency (amount of ribosome footprints mapped on the ORF was divided by amount of RNA reads mapped on the ORF), but not amount of the mapped ribosome footprints, was examined.

The results showed that the second ORFs of seven the eight selected bicistronic RTs, the exception being RT7, were rarely translated in contrast to first ORFs (Fig. 3b). This indicates that translational re-initiation of second ORFs rarely occurs in bicistronic RTs. Taking the results from Figs. 2 and 3b into account, it is assumed that termination codons and the downstream regions of the first ORF are recognized by the NMD machinery as premature termination codons and long 3′ UTRs, which are well-known NMD triggers.

To get further insight into the relationship between the translational state of second ORFs and NMD, RTFs (RT2F and RT5F) harboring a fused ORF were expressed in leaves of *Nicotiana benthamiana*; the termination codon of the first ORF and internal UTR were removed from the RTs. Accumulation of both RTFs was higher than that of RTs (Fig. 3c) and the translation level of the second ORF region of the RTFs was compatible with that of the first ORFs (Fig. 3d and Supplementary Fig. 4). RNAi assays against the *NbUPF1* gene by co-transfection with double-stranded RNA of a partial *NbUPF1* sequence showed relatively higher accumulation of RTs but not RTFs compared with the control in which double-stranded RNA of a partial sequence of the Venus fluorescence gene was co-transfected (Fig. 3e). These results indicate that RTFs are not subject to NMD (Fig. 3e) and, as a result, stabilized (Fig. 3c). In summary, termination codon of the first ORF is associated with NMD.

### pre-RTs carrying IS introns are not accumulated under a splicing inhibitor

**Fig. 4:**
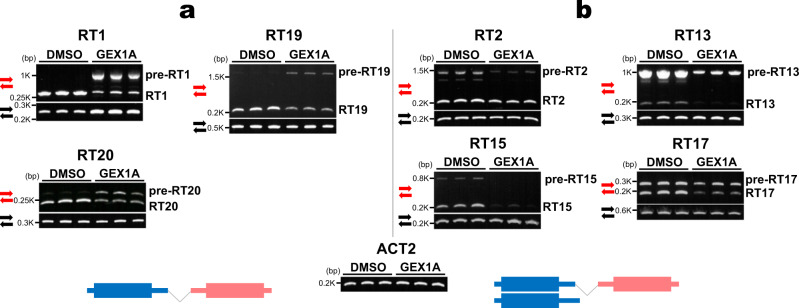
Splicing inhibitor treatment assay in *Arabidopsis* seedlings. The results were divided into two specific types, **a** and **b**. *n* = 3 biologically independent samples. **a** Pre-RTs undergoing intron retention increased but mature RTs decreased under the inhibitor (RT1, RT19 and RT20). **b** Both pre-RTs and mature RTs decreased (RT2, RT13, RT15 and RT17). The red primer pair amplifies the IS intron-containing joint region between the first and second genes, while the black primer pair amplifies the intron-less region of the first genes. Three biological replicates were performed. *ACT2* was used as an internal control. The difference between the two types, **a** and **b**, is shown in the lower schematic.

As exemplified in Fig. 1a, ISs were detected in 158 RTs. To investigate the effect of IS on accumulation of mature RTs, young dark-grown WT seedlings were treated with a splicing inhibitor, GEX1A^31^, and accumulation of RTs and pre-RTs that still carry IS introns was detected by semi-quantitative RT-PCR. Importantly, the results can be classified into two specific types; (a) pre-RTs carrying introns were increased but mature RTs were decreased under the inhibitor (RT1, RT19, and RT20 in Fig. 4a) and (b) both pre-RTs and mature RTs were decreased (RT2, RT13, RT15, and RT17 in Fig. 4b).

The apparent characteristic differences between the two are that the former RT loci dominantly produce only RTs, while the latter loci produce both monocistronic mRNAs for the first gene and RTs, and that, in the latter type, polyadenylation sites (3′ end) of the first monocistronic mRNAs were detected inside the IS introns as shown in Fig. 1a. Transcription termination of the first monocistronic mRNAs, that occurs inside an IS intron region, was seen in 106 of the 158 RT loci undergoing IS by ISO-seq analysis (Supplementary Data 1).

### ISs promote accumulation of RTs

**Fig. 5:**
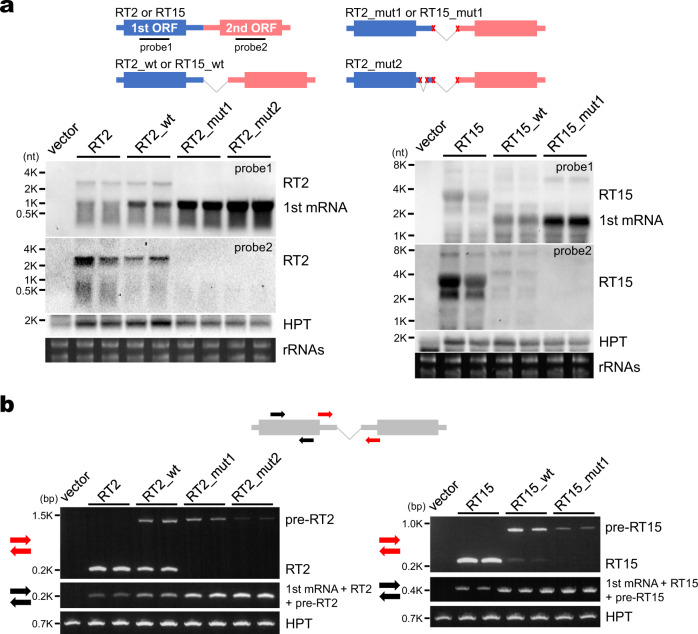
Northern blot and RT-PCR analyses for RTs, pre-RTs, and pre-RTs with mutations at splicing sites by transient expression in *Nicotiana benthamiana*. **a** Northern blot analysis detecting mature RTs and mRNAs generated from the first genes of the RT2 and RT15 loci. In RT2_mut1, the donor (GUA) and acceptor (CAG) sites of the major IS intron were replaced with CUC and GGG, respectively, and, in RT2_mut2, the donor (GUA) and acceptor (CAG) sites of both the major and minor introns were replaced with CUC and GGG, respectively. In RT15_mut1, the donor (GUA) and acceptor (AAG) sites were replaced with CUC and GGG, respectively. The upper illustrations indicate the transiently expressed RT cassettes. *n* = 2 biologically independent samples. **b** RT-PCR analysis for the detection of mature RTs and pre-RTs. The upper illustration indicates the positions of the two primer sets used. The red primer pair amplifies the pre-RT and mature RT separately, while the black primer pair redundantly amplifies the mRNA of the first gene (1st mRNA), the mature RT and the pre-RT as a single band. *n* = 2 biologically independent samples. HPT = hygromycin phosphotransferase.

Considering the above result, we speculated that ISs eliminate the chance of transcription termination of the first mRNAs and promote accumulation of RTs. To verify this possibility, pre-RTs carrying IS introns and mutated pre-RTs in which both splicing donor and acceptor sites are replaced with other sequences were transiently expressed in *Nicotiana benthamiana* leaves and detected by Northern blot analysis (Fig. 5a). RT2 and RT15 were selected for the analysis. Since, in the RT2 locus, two IS introns, major and minor weak ones, were detected (Fig. 1a), two mutated pre-RTs were used for the analysis. The results showed that, in both the RT2 and RT15 series, increased accumulation of the first monocistronic mRNAs in the mutated pre-RTs (RT_mut1 and RT_mut2) was detected compared with that in the wild-type pre-RTs (RT_wt).

However, pre-RTs in some samples were not detected in the Northern analysis. In order to detect these, RT-PCR analysis was also performed using the same RNA samples and showed that, in both the RT2 and RT15 series, accumulation of pre-RTs in the mutated pre-RTs was lower than that in the wild-type ones (Fig. 5b).

It was assumed that IS eliminates not only polyadenylation sites of the first genes but also the polyadenylation signals. We searched for *Arabidopsis* polyadenylation signals and cleavage elements near the polyadenylation sites of the first genes, which have been reported previously^25–27^. Of the 106 RTs undergoing IS, 86 possess polyadenylation signal-like hexamers and 86 have cleavage element-like hexamers in the 3′ region from −150 nt or donor sites to polyadenylation sites of first genes (Supplementary Data 1 and Supplementary Fig. 5). Taken together with Fig. 4 and Fig. 5, this result suggests that IS events diminish the chance of transcription termination in the first gene by elimination of polyadenylation signals and sites.

## Discussion

**Fig. 6:**
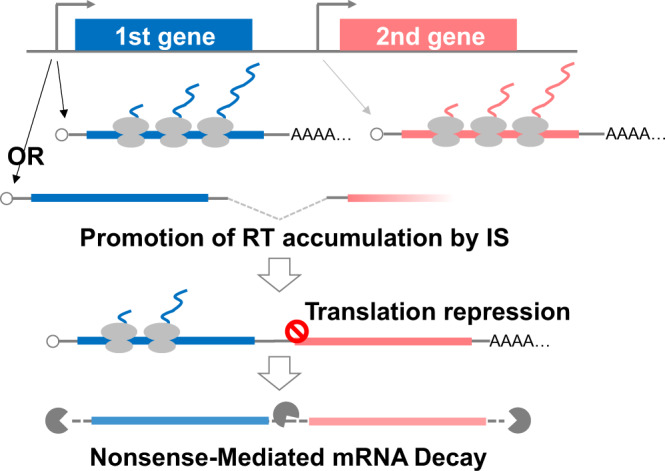
Model for suppression of IS-stimulated readthrough transcription by NMD. IS events prevent transcription termination at the 3' end of the first gene and promote readthrough transcriptional elongation and accumulation of RTs. However, NMD selectively degrades them by recognizing termination codons of first ORFs and downstream UTRs including second ORFs as premature termination codons and long 3' UTRs.

The molecular mechanisms for the biogenensis and metabolism of RTs have not yet been elucidated. In this report, we have identified RTs throughout the young *Arabidopsis* seedling genome and examined their structural features and fate. In many RT loci, polycistronic RTs are co-transcribed with monocistronic mRNAs. Remarkably, IS events are largely detected in the joint regions between first and second genes (Fig. 1). Splicing inhibitor treatment (Fig. 4) and transient expression assays of mutated pre-RTs (Fig. 5) suggested that IS events prevent transcription termination at the 3′ end of the first gene by eliminating polyadenylation signals and sites, which can promote readthrough transcriptional elongation and accumulation of RTs (Fig. 6). However, there remain many loci where readthrough transcription occurs independently of IS. It is possible that transcription termination activity is originally weak in the first genes of these loci. A previous report showed that, in human, U1 small nuclear ribonucleoprotein (snRNP), a component of spliceosome, functions not only to control splicing but also to protect transcribing pre-mRNAs from premature transcription termination^32^. It is possible that a similar mechanism could positively act on read-through transcription by preventing transcription termination at the 3′ end of the first gene independent of IS, although there have been no reports of U1 snRNP functioning as a protector in plants. It should be important to examine the relationship between U1 snRNP and read-through transcription.

In addition, and importantly, most of the RTs accumulated more in *upf1* and *upf3* NMD-deficient mutants than in WT (Table 1 and Fig. 2), indicating that RTs are NMD targets. Ribo-seq analysis for transiently expressed RTs showed that second ORFs on RTs are rarely translated in contrast to the first ORFs, probably because of difficulty in translation re-initiation at the second ORFs (Fig. 3). These observations suggest that termination codons of first ORFs and downstream untranslated regions including second ORFs are recognized as premature termination codons and long 3′ UTRs, respectively, which are strong NMD triggers^2^. In some mRNA variants, upstream ORFs are located in front of the protein-coding main ORFs. The relationship between the first and second ORFs is analogous to that between upstream ORFs and main ORFs, since upstream ORFs also inhibit translation of main ORFs and cause degradation by NMD^33^.

RNA splicing can be an indirect determinant of RNA fate. Alternative splicing events often give protein diversity, while it has also been reported that alternative splicing incidentally produces premature termination codons and/or relatively longer 3′ UTRs, triggering NMD^2,7^. Furthermore, as described above, it is supposed that the common termination codon of the first gene becomes a premature termination codon-like element by undergoing readthrough transcription and IS, another type of splicing. Thus, we present a novel link between RNA splicing and NMD.

In prokaryotic transcription termination, both hairpin RNA structures and U-rich sequences located at the 3′UTR weaken physical interaction between the RNA polymerase and the DNA template, and cause detachment of the polymerase from the template^34^. However, the eukaryotic termination mechanism is different and requires endonucleolytic cleavage and polyadenylation at the 3′ end of the transcribing mRNA. Our data suggest that genes of prokaryotic origin are likely to undergo readthrough transcription (Fig. 1b). It is easy to speculate that they have not yet acquired sufficient elements for eukaryotic transcription termination, such as polyadenylation signals, following their transition from organellar to nuclear genes and, consequently, readthrough transcription is likely to occur. Particularly in plants, polyadenylation signals are very various and complex, because usage of the most frequently used polyadenylation signal, AAUAAA, is still low^25^. Although it is difficult to identify apparent plant polyadenylation signals in the 3′ region of the first genes of the RT loci, many ISs can probably eliminate either one or both of the polyadenylation signals and endonucleolytic cleavage sites (Supplementary Fig. 5). In addition, stalling of RNA polymerase II downstream of the polyadenylation site is an excellent indicator of transcription termination^35^. It is possible that RNA polymerase II stalling is less likely to occur at the transcription termination site of the first gene of RTs. This remains to be investigated in the future. In summary, it is apparent that RTs have arisen from genes whose transcription termination is relatively weak or attenuated by IS.

Nuclear genes encoding organelle-localized proteins, represented by *PPR* and *TPR*, undergo transcriptional readthrough and their expression is properly regulated by NMD (Fig. 1b). However, it is speculated that, in *upf1* mutants, excessive protein production from RTs that have escaped degradation might disrupt organelle function to some extent and be one of the causes of the morphological abnormalities and pale green phenotype reported previously^8,10^.

Intronic polyadenylation (IPA) occurs inside an intron of a gene comprising of a single protein-coding ORF and may expand protein diversity by producing a truncated one^36–38^. IPA events in humans are associated with diseases like cancer^37,38^. In *Arabidopsis*, it has been reported that hypoxia stress increases the number of mRNAs with IPA and the abnormal mRNAs with IPA may be relatively unstable^36^. Both splicing of introns containing IPA sites and IS are similar systems for eliminating polyadenylation. However, IPA splicing produces normal mRNAs with canonical 3′ UTRs, while, conversely, IS of an intergenic intron-containing canonical polyadenylation sites produces RTs that are identified as abnormal transcripts by NMD.

Most of the bicistronic RTs examined are accumulated more in *upf* mutants compared with WT (Fig. 2). However, there were two RTs that were most likely insensitive to NMD under our physiological conditions. Of the insensitive ones, RT11 was previously reported to be accumulated in *upf* mutants under different conditions from those in this report^21^. The other insensitive one, RT7, is strongly induced upon blue-light irradiation after darkness unlike the other RTs examined (Fig. 2), because the first gene, which is responsible for the transcriptional induction, encodes a DNA photolyase protein that may be required for repairing light-induced DNA damage^39^. Taken together, it is speculated that plant cells incipiently equip any as yet unknown systems for alleviating the opportunity for RNA decay, which act dependently in any growth period or under any physiological conditions. They remain to be determined in the future. We consider that translational regulatory elements, such as the internal ribosome entry sites located in front of the start codons of the second ORFs, may be candidates.

We have investigated the biogenensis and metabolism of polycistronic RTs, using not only ISO-seq but also CAGE and Ribo-seq, and have discovered a novel aspect of gene expression regulation involved in RTs. As exemplified in this work, emerging integrated omics strategies using long-read sequencing technologies will make it easier to find unknown regulatory mechanisms for gene expression and reveal their involvement in multiple biological phenomena.

## Methods

### Plant and growth conditions

Wild type, *upf1-1*^10^ and *upf3-1* (*Arabidopsis* ecotype Col-0)^9,40^ were used in this research. Seed sterilization, sowing and growth conditions in darkness and blue light have been described previously^4^, with the exception that the blue-light intensity here was 9 μmol m^−2^ s^−1^.

### CAGE, RNA-seq, ISO-seq, and Nanopore sequencing

The total RNA used for CAGE, RNA-seq, ISO-seq and Nanopore sequencing was extracted using Plant RNeasy Mini Kits (Qiagen) from 3-day-old seedlings grown under darkness and then irradiated with blue light for 3 h. Library construction for CAGE and RNA-seq was carried out using the CAGE Library Preparation Kit (DNAFORM) and TruSeq Stranded mRNA Preparation Kit (Illumina), respectively. The libraries (three replicates) were sequenced by HiSeq4000 or HiSeqX platforms. ISO-seq libraries were constructed with the SMARTer® PCR cDNA Synthesis Kit (Clontech) and SMRTbell Express Template Prep Kit 2.0 (Pacific Bioscience, Menlo Park, CA, USA) and sequenced by a PacBio Sequel II Platform. Nanopore libraries were constructed using PCR-cDNA Sequencing Kits (SQK-PCS109, Oxford Nanopore) and sequenced by the MinION platform.

CAGE reads were mapped to the TAIR10 genome using both BWA v0.7.12^41^ and HISAT2 v2.0.5^42^. Merged mapped reads were counted with CAGEr software v1.16.0^43^ and normalized with tags per million (TPM).

After removing low quality reads (QV < 20), RNA-seq data were mapped onto The Arabidopsis Information Resource (TAIR) 10 genome with STAR version 020201^44^. FPKM normalization was implemented with Cufflinks v2.2.1^45^.

ISO-seq data were processed using SMRT Link ver. 9.0.0 (PacBio) following their ISO-seq protocols with default parameters. First, consensus sequences were generated from subread data with ccs, then primer sequences and demultiplex were removed with the Lima program. Quality control, clustering and polishing were performed with IsoSeq3 and the processed data were mapped to the TAIR10 genome with pbmm2. BUSCO v5.1.3 and the eudicots_odb10 dataset^46^ were used to estimate the completeness of the ISO-seq data.

Nanopore reads were generated with EPI2ME Desktop Agent v3.4.2 (Oxford Nanopore) that analyzes the raw electrical signals into FAST5 files and converts them to FASTQ files. The raw sequences were mapped to the *Arabidopsis* genome (TAIR10) using minimap2 version 2.20-r1064-dirty^47^ with the following parameters: -ax splice -ub -k14 -G1000. The mapped data were sorted and converted into gff files using samtools version 1.6 and spliced_bam2gff version 1.2, respectively.

These next-generation sequencing data were visualized using JBrowse 1.16.11^48^.

Information on protein subcellular localization and chromosome distribution were derived from the deposited data and tool in TAIR (https://www.arabidopsis.org/), respectively.

### Construction of plasmids for RT transient expression

Full-length RT sequences were artificially synthesized by Eurofins Genomics K.K. (Tokyo, Japan) and amplified by PCR using the primers (HiFi_F and HiFi_R) listed in Supplementary Table 1. PCR products were cloned into the Xba I-Not I cloning site, which is between the CaMV 35 S promoter and NOS terminator, of the pSK1 vector^49^ using the NEBuilder HiFi DNA Assembly Cloning Kit (NEB).

To create the IS wild-type intron and mutated intron-containing constructs, the 5′ regions, IS introns, and 3′ regions of RT2 and RT15 were amplified by PCR using the primers listed in Supplementary Table 1 (HiFi_F and 5′exon_R, wt_IS_intron_F and wt_IS_intron_R or mut1_IS_intron_F and mut1_IS_intron_R, and 3′exon_F and HiFi_R, respectively). The templates used were the RT clone for the amplification of the 5′ and 3′ regions, and genomic DNA for the amplification of the IS intron. The three fragments were integrated and inserted into the pSK1 vector using the NEBuilder HiFi DNA Assembly Cloning Kit. For the RT2 mutant2, a second round of PCR amplification was performed with the RT2_mut1 plasmid as a template and the primers listed in Supplementary Table 1 (HiFi_F and 5′exon2_R for the 5′ region, mut2_minor_IS_intron_F and mut2_minor_IS_intron_R for the mutated minor IS intron, and 3′exon2_F and HiFi_R for the 3′ region). These three fragments were also integrated and inserted into the pSK1 vector as described above.

To create the RT2F and RT5F constructs, the first half and second half of them were amplified by PCR using the primers listed in Supplementary Table 1 (HiFi_F and 1st_R, and 2nd _F and HiFi_R, respectively). The two fragments were integrated and inserted into the pSK1 vector.

To create the dsUPF1 construct, the *NbUPF1* fragment was amplified from a cDNA library using the primers listed in Supplementary Table 1 (dsUPF1_F and dsUPF1_R) and subcloned into the pENTR TOPO vector (Invitrogen). The fragment was inserted into two inverted sites of pBI-sense, antisense-GW vector (IN3-VEC3, Inplanta Innovations, Inc.). The dsVenus plasmid has been described previously^50^.

### Transient expression assay in *Nicotiana benthamiana*

The plasmids constructed were transformed into Agrobacterium strain GV3101. The Agrobacterium was cultured in LB medium containing 50 μg/ml kanamycin at 29 °C for 20 h. The cells were pelleted by centrifugation, resuspended in infiltration buffer (10 mM MES pH 5.8, 10 mM MgCl_2_, 100 μM acetosyringone) to an OD_600 nm_ of 1.0 and incubated on the bench for 2 h. For Ribo-seq analysis, bacteria harboring RT1, RT2, RT3 and RT5 (set A), RT6, RT7, RT9 and RT11 (set B), and RT2F and RT5F (set F) were mixed equally in each set for co-expression. The bacterial solution was infiltrated by syringe into holes on the leaves of one-month-old *Nicotiana benthamiana* plants. Three days after infiltration, the leaves were harvested and used for Ribo-seq, Northern blot and semi-quantitative RT-PCR analyses but not RNAi assays.

### Ribo-seq

Sample preparation and library construction for Ribo-seq (ribosome profiling) and RNA-seq have been described previously^4^. The libraries were sequenced using HiSeq4000 or HiSeqX platforms. Two replicates for *Arabidopsis* wild-type and *upf1-1* seedlings and three replicates for the transient assay (sets A, B, and F) were performed.

After removing rRNA and tRNA reads with Bowtie version 2.3.4.1^51^, Ribo-seq reads were mapped to the TAIR10 genome with TopHat version 2.1.1^45^. The footprint length of 27–29 nt was applied for normalizing read counts by DESeq 1.42.0 in an R package^52^. Translation efficiency of each ORF was calculated by dividing the number of ribosomal footprints (derived from Ribo-seq) mapped to each ORF by the number of RNA reads (derived from RNA-seq) mapped to each ORF region.

### GEX1A treatment

GEX1A (herboxidiene, 10 mM stock in DMSO, Focus Biomolecules) was used for the splicing inhibition assay^31^. Dark-grown 3-day-old seedlings were soaked in GEX1A solution (5 μM GEX1A, 10 mM MES pH 5.8) for 6 h and then sampled for semi-quantitative RT-PCR.

### Semi-quantitative RT-PCR for *Arabidopsis* samples

Total RNA was extracted from seedlings using NucleoSpin® RNA Plant (Takara Bio.) which includes DNase I treatment. Reverse transcription was performed using a PrimeScript II 1st strand cDNA Synthesis Kit (Takara Bio.), and PCR was performed using KOD One® PCR Master Mix (TOYOBO) and the RT product as a template. Thirty-five cycles of PCR were performed for detection of RTs and 30 cycles for detection of the first genes in 20 μl reaction volume. Primers used for PCR are listed in Supplementary Table 2. PCR products were loaded onto a 1.5% agarose gel and electrophoresed in TBE buffer followed by detection under ultra-violet (UV) light using GelRed stain (Nacalai Tesque, Japan). As size markers, Gene Ladder 100 (Nippon Gene) or GD 1Kb plus DNA ladder RTU (GeneDireX) were used.

### Northern blot analysis and semi-quantitative RT-PCR for transient expression assays

Total RNA was extracted from the agroinfiltrated leaves of *Nicotiana benthamiana* using Trizol reagent (ThermoFisher). Ten micrograms of total RNA were loaded onto a denaturing 1% agarose gel, electrophoresed in MOPS buffer, and blotted onto Hybond N + membrane (GE Healthcare) by the capillary method in 20x SSC. Dynamarker Prestain Marker for RNA High (BioDynamics Laboratory Inc.) was used as a size marker. The membrane was cross-linked by UV exposure (70,000 J/cm^2^), rinsed in 3x SSC, and dried. The DNA fragment containing the T7 promoter sequence was amplified by PCR using the primers listed in Supplementary Table 3. DIG-labeled probes were constructed by T7 RNA polymerase reverse transcription using a DIG Northern Starter Kit (Merck). Hybridization was performed at 68 °C in DIG Easy Hyb buffer containing the probes. The membrane was washed two times with 2 × SSC, 0.1% SDS for 20 min at room temperature and then 3 times with 0.1 × SSC, 0.1% SDS for 20 min at 68 °C. Detection of signals was performed according to the manufacturer’s instructions.

For RT-PCR, total RNA was digested with Turbo DNase I (ThermoFisher). RT-PCR, gel electrophoresis, and detection were performed as described above.

### RNAi assay against *NbUPF1*

After resuspension in infiltration buffer and density adjustment, bacteria harboring RT2 and RT5, and RT2F and RT5F were mixed equally and then subsequently mixed with bacteria harboring dsUPF1 or dsVenus at a ratio of 3:7. Six days after infiltration the leaves were harvested and used for semi-quantitative RT-PCR analyses using the primers listed in Supplementary Table 4.

### Statistics and reproducibility

The number of biological replicates is indicated in the legend of each figure. Significant differences in translation efficiency between multiple ORF regions were determined by Tukey’s test. Significant enrichment of the genes encoding mitochondria or chloroplast-localized proteins in the first genes of the RT loci was determined by hypergeometric test.

### Reporting summary

Further information on research design is available in the Nature Portfolio Reporting Summary linked to this article.

## Supplementary information

Supplementary Information

Description of Additional Supplementary Files

Supplementary Data 1

Supplementary Data 2

Supplementary Data 3

Reporting Summary

## Data Availability

The dataset of sequenced reads by the next-generation sequencers is deposited in the DDBJ/EMBL/GenBank BioProject under accession number DRA014187. Source data of Figs. 3b and 3d are provided in Supplementary Data 2. The Addgene IDs of newly-constructed plasmids were available in Supplementary Data 3. Uncropped data are shown as Supplementary Fig. 6 at the end of Supplementary Information.

## References

[1] Chantarachot T, Bailey-Serres J (2018). Polysomes, stress granules, and processing bodies: a dynamic triumvirate controlling cytoplasmic mRNA fate and function. Plant Physiol..

[2] Ohtani M, Wachter A (2019). NMD-based gene regulation—a strategy for fitness enhancement in plants?. Plant Cell Physiol..

[3] Kurihara Y (2009). Genome-wide suppression of aberrant mRNA-like noncoding RNAs by NMD in Arabidopsis. Proc. Natl Acad. Sci. USA.

[4] Kurihara Y (2018). Transcripts from downstream alternative transcription start sites evade uORF-mediated inhibition of gene expression in. Proc. Natl Acad. Sci. USA.

[5] Nyikó T, Sonkoly B, Mérai Z, Benkovics AH, Silhavy D (2009). Plant upstream ORFs can trigger nonsense-mediated mRNA decay in a size-dependent manner. Plant Mol. Biol..

[6] Uchiyama-Kadokura N (2014). Polyamine-responsive ribosomal arrest at the stop codon of an upstream open reading frame of the AdoMetDC1 gene triggers nonsense-mediated mRNA decay in Arabidopsis thaliana. Plant Cell Physiol..

[7] Drechsel G (2013). Nonsense-mediated decay of alternative precursor mRNA splicing variants is a major determinant of the Arabidopsis steady state transcriptome. Plant Cell.

[8] Arciga-Reyes L, Wootton L, Kieffer M, Davies B (2006). UPF1 is required for nonsense-mediated mRNA decay (NMD) and RNAi in Arabidopsis. Plant J..

[9] Hori K, Watanabe Y (2005). UPF3 suppresses aberrant spliced mRNA in Arabidopsis. Plant J..

[10] Yoine M, Ohto MA, Onai K, Mita S, Nakamura K (2006). The lba1 mutation of UPF1 RNA helicase involved in nonsense-mediated mRNA decay causes pleiotropic phenotypic changes and altered sugar signalling in Arabidopsis. Plant J..

[11] Merchante C (2015). Gene-specific translation regulation mediated by the hormone-signaling molecule EIN2. Cell.

[12] Raxwal VK (2020). Nonsense-mediated RNA decay factor UPF1 is critical for posttranscriptional and translational gene regulation in Arabidopsis. Plant Cell.

[13] Salgado H, Moreno-Hagelsieb G, Smith TF, Collado-Vides J (2000). Operons in *Escherichia coli*: genomic analyses and predictions. Proc. Natl Acad. Sci. USA.

[14] Jia J (2020). Post-transcriptional splicing of nascent RNA contributes to widespread intron retention in plants. Nat. Plants.

[15] Li R (2020). Direct full-length RNA sequencing reveals unexpected transcriptome complexity during. Genome Res.

[16] Mo W (2021). Landscape of transcription termination in Arabidopsis revealed by single-molecule nascent RNA sequencing. Genome Biol..

[17] Parker, M. T. et al. Nanopore direct RNA sequencing maps the complexity of Arabidopsis mRNA processing and m6A modification. *Elife* **9**, e49658 (2020). 10.7554/eLife.49658 PMC6959997 31931956

[18] Thomas QA (2020). Transcript isoform sequencing reveals widespread promoter-proximal transcriptional termination in Arabidopsis. Nat. Commun..

[19] Wang K (2019). Multi-strategic RNA-seq analysis reveals a high-resolution transcriptional landscape in cotton. Nat. Commun..

[20] Gallaher, S. D. et al. Widespread polycistronic gene expression in green algae. *Proc. Natl Acad. Sci. USA* **118**, e2017714118 (2021). 10.1073/pnas.2017714118 PMC7896298 33579822

[21] Lorenzo-Orts L (2019). Concerted expression of a cell cycle regulator and a metabolic enzyme from a bicistronic transcript in plants. Nat. Plants.

[22] Crisp PA (2018). RNA polymerase II read-through promotes expression of neighboring genes in SAL1-PAP-XRN retrograde signaling. Plant Physiol..

[23] Kurihara Y (2012). Surveillance of 3’ noncoding transcripts requires FIERY1 and XRN3 in Arabidopsis. G3 (Bethesda).

[24] Krzyszton M (2018). Defective XRN3-mediated transcription termination in Arabidopsis affects the expression of protein-coding genes. Plant J..

[25] Loke JC (2005). Compilation of mRNA polyadenylation signals in Arabidopsis revealed a new signal element and potential secondary structures. Plant Physiol..

[26] Shen Y (2008). Genome level analysis of rice mRNA 3’-end processing signals and alternative polyadenylation. Nucleic Acids Res.

[27] Wu X (2011). Genome-wide landscape of polyadenylation in Arabidopsis provides evidence for extensive alternative polyadenylation. Proc. Natl Acad. Sci. USA.

[28] Shiraki T (2003). Cap analysis gene expression for high-throughput analysis of transcriptional starting point and identification of promoter usage. Proc. Natl Acad. Sci. USA.

[29] Fujita T, Kurihara Y, Iwasaki S (2019). The plant translatome surveyed by ribosome profiling. Plant Cell Physiol..

[30] Merchante C, Stepanova AN, Alonso JM (2017). Translation regulation in plants: an interesting past, an exciting present and a promising future. Plant J..

[31] AlShareef S (2017). Herboxidiene triggers splicing repression and abiotic stress responses in plants. BMC Genomics.

[32] Kaida D (2010). U1 snRNP protects pre-mRNAs from premature cleavage and polyadenylation. Nature.

[33] Kurihara, Y. uORF shuffling fine-tunes gene expression at a deep level of the process. *Plants* **9**, 608 (2020). 10.3390/plants9050608 PMC7284334 32403214

[34] Henkin TM (1996). Control of transcription termination in prokaryotes. Annu Rev. Genet..

[35] Kindgren P, Ivanov M, Marquardt S (2020). Native elongation transcript sequencing reveals temperature dependent dynamics of nascent RNAPII transcription in Arabidopsis. Nucleic Acids Res..

[36] de Lorenzo L, Sorenson R, Bailey-Serres J, Hunt AG (2017). Noncanonical alternative polyadenylation contributes to gene regulation in response to hypoxia. Plant Cell.

[37] Zhao Z (2021). Comprehensive characterization of somatic variants associated with intronic polyadenylation in human cancers. Nucleic Acids Res..

[38] Zhao Z (2021). Cancer-associated dynamics and potential regulators of intronic polyadenylation revealed by IPAFinder using standard RNA-seq data. Genome Res..

[39] Zhang M, Wang L, Zhong D (2017). Photolyase: dynamics and electron-transfer mechanisms of DNA repair. Arch. Biochem. Biophys..

[40] Alonso JM (2003). Genome-wide insertional mutagenesis of Arabidopsis thaliana. Science.

[41] Li H, Durbin R (2009). Fast and accurate short read alignment with Burrows-Wheeler transform. Bioinformatics.

[42] Zhang, Y., Park, C., Bennett, C., Thornton, M. & Kim, D. Rapid and accurate alignment of nucleotide conversion sequencing reads with HISAT-3N. *Genome Res.* **31**, 1290–1295 (2021). 10.1101/gr.275193.120 PMC8256862 34103331

[43] Haberle V, Forrest AR, Hayashizaki Y, Carninci P, Lenhard B (2015). CAGEr: precise TSS data retrieval and high-resolution promoterome mining for integrative analyses. Nucleic Acids Res..

[44] Dobin A (2013). STAR: ultrafast universal RNA-seq aligner. Bioinformatics.

[45] Trapnell C (2012). Differential gene and transcript expression analysis of RNA-seq experiments with TopHat and Cufflinks. Nat. Protoc..

[46] Manni M, Berkeley MR, Seppey M, Simão FA, Zdobnov EM (2021). BUSCO update: novel and streamlined workflows along with broader and deeper phylogenetic coverage for scoring of eukaryotic, prokaryotic, and viral genomes. Mol. Biol. Evol..

[47] Li H (2018). Minimap2: pairwise alignment for nucleotide sequences. Bioinformatics.

[48] Buels R (2016). JBrowse: a dynamic web platform for genome visualization and analysis. Genome Biol..

[49] Kojima S (1999). A binary vector plasmid for gene expression in plant cells that is stably maintained in Agrobacterium cells. DNA Res..

[50] Kurihara Y (2015). Polycistronic expression of RNA silencing suppressor protects its own mRNA from RNA silencing. Plant Biotechnol..

[51] Langmead B, Salzberg SL (2012). Fast gapped-read alignment with Bowtie 2. Nat. Methods.

[52] Anders S, Huber W (2010). Differential expression analysis for sequence count data. Genome Biol..

