# Supplementary material for: Intergenic splicing-stimulated transcriptional readthrough is suppressed by nonsense-mediated mRNA decay in Arabidopsis
Source: Commun Biol. 2022 Dec 20;5:1390. doi: 10.1038/s42003-022-04348-y (PMC9768141; doi:10.1038/s42003-022-04348-y)
Supplement: Supplementary file 1 — Supplementary Information [file 42003_2022_4348_MOESM1_ESM.pdf]

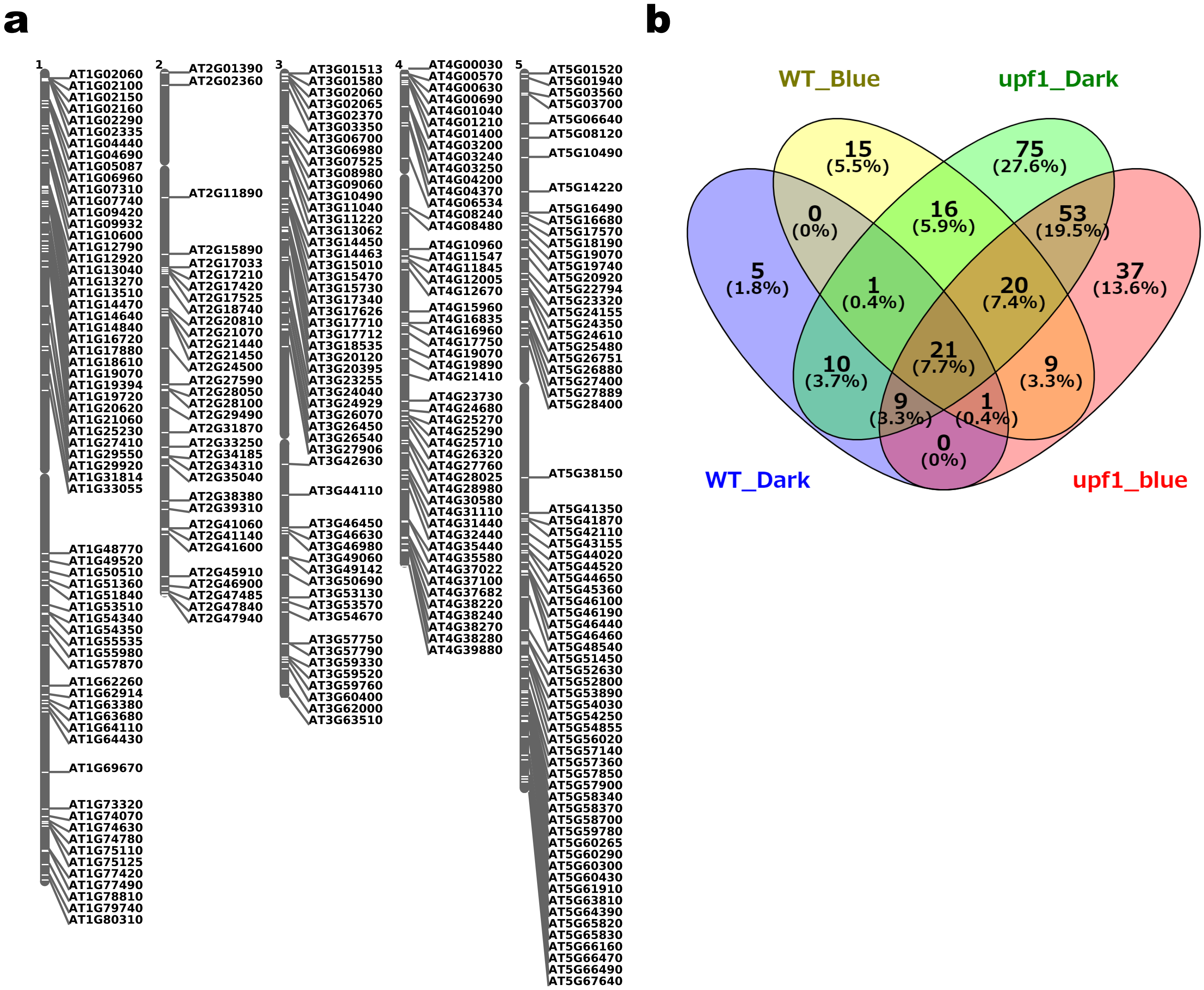

Supplementary Fig. 1 **a** Chromosomal distribution of the 271 RT loci. **b** Overlap of the RT loci detected under the four conditions tested.

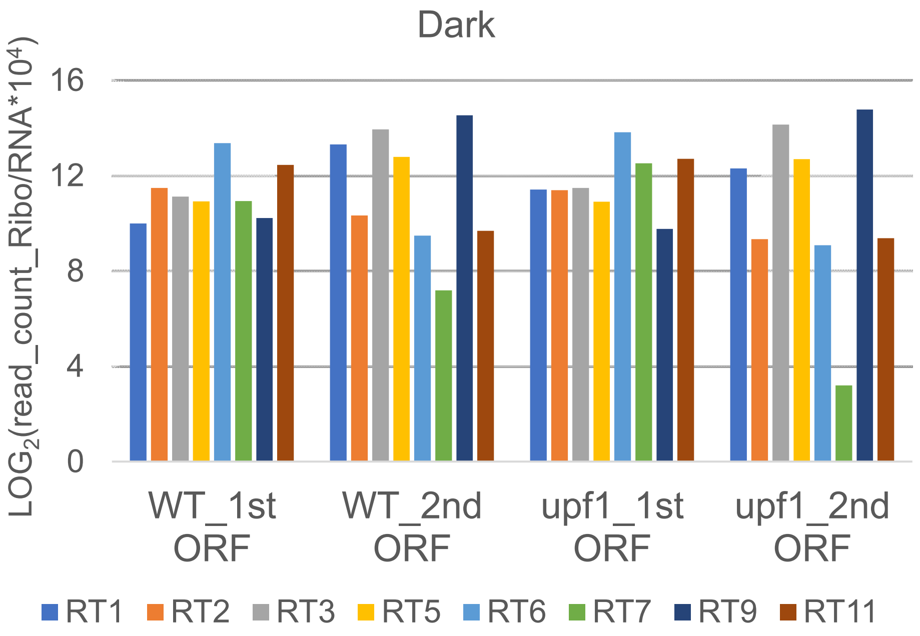

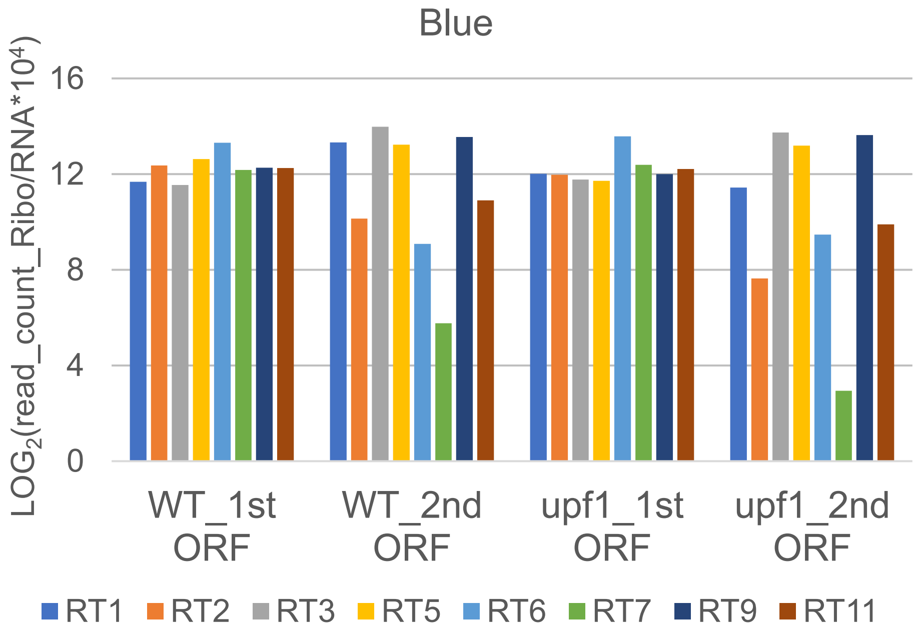

Supplementary Fig. 2 Ribo-seq analysis for the first and second genes of RT1, RT2, RT3, RT5, RT6, RT7, RT9 and RT11 in *Arabidopsis*. Translation efficiency of each ORF is shown.

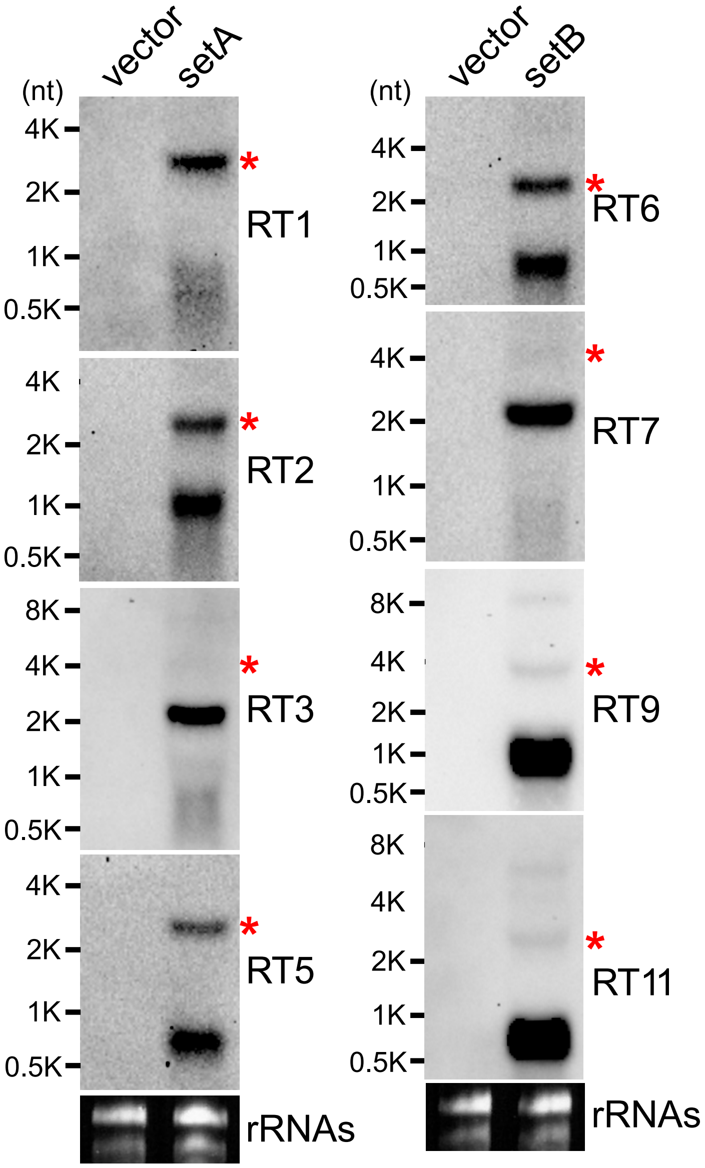

Supplementary Fig. 3 Northern blot analysis for transiently expressed RTs in *Nicotiana benthamiana* leaves. Red asterisks indicate deduced positions of full-length RTs.

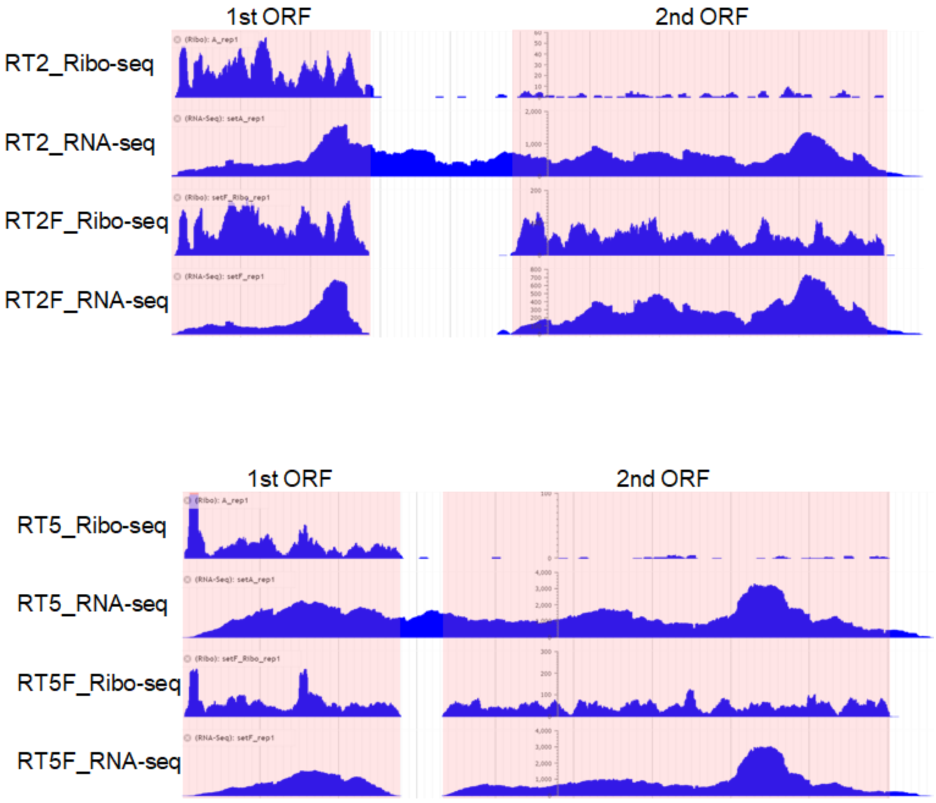

Supplementary Fig. 4 Genome browser view of RT2 and RT5 loci. Each ORF is highlighted by pink color.

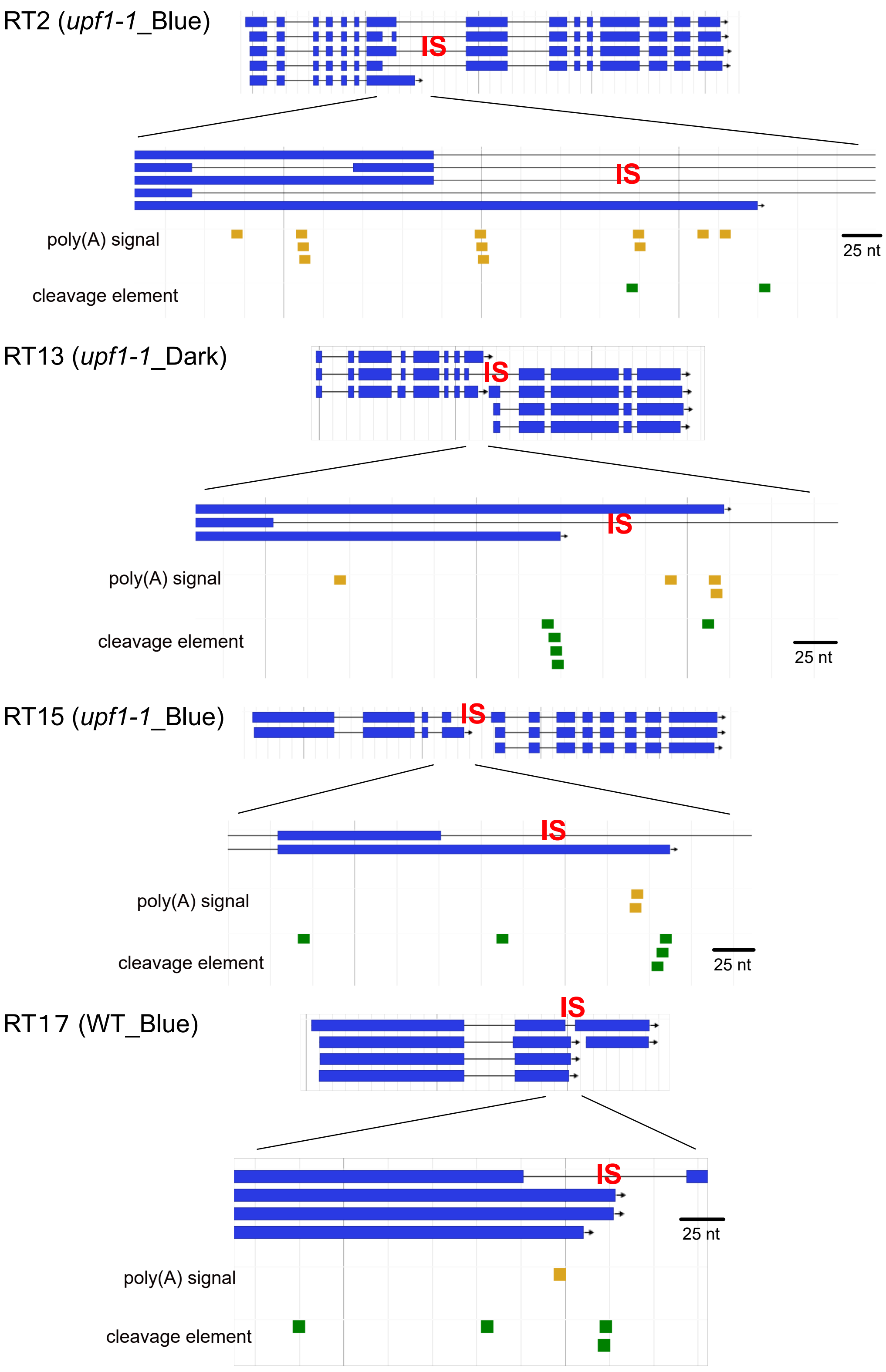

Supplementary Fig. 5 Examples of positions of polyadenylation signal-like and cleavage element-like hexamers in IS introns. Polyadenylation signal-like hexamers; AAUAAA, AUAAAA, UAAUAA, AAAUAA, AUAAAU, AUAAUA, AUAUAU, UAUAUA, AAUAAU, UAUAAA. Cleavage element-like hexamers; UUUUUA, UUUCUU, UGUUUA, UUGUUU, UUCUUA, UUUCUA, UUUUCA, UUCUUU, UUAUUA, UUUUCU. They were selected with reference to Loke *et al.* (2005) Plant Physiol. 138: 1457-1468.

Supplementary Figure 6 Uncropped data.

Used markers (M1 and M2) for agarose gel electrophoresis

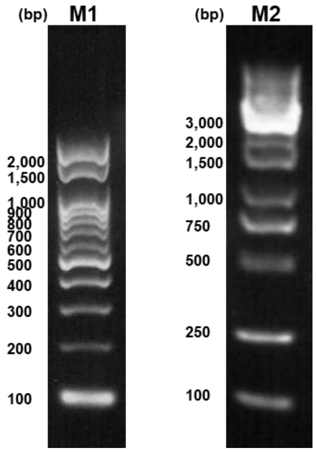

Marker (M1 or M2) is indicated on each image.

Unprocessed data for Fig. 2

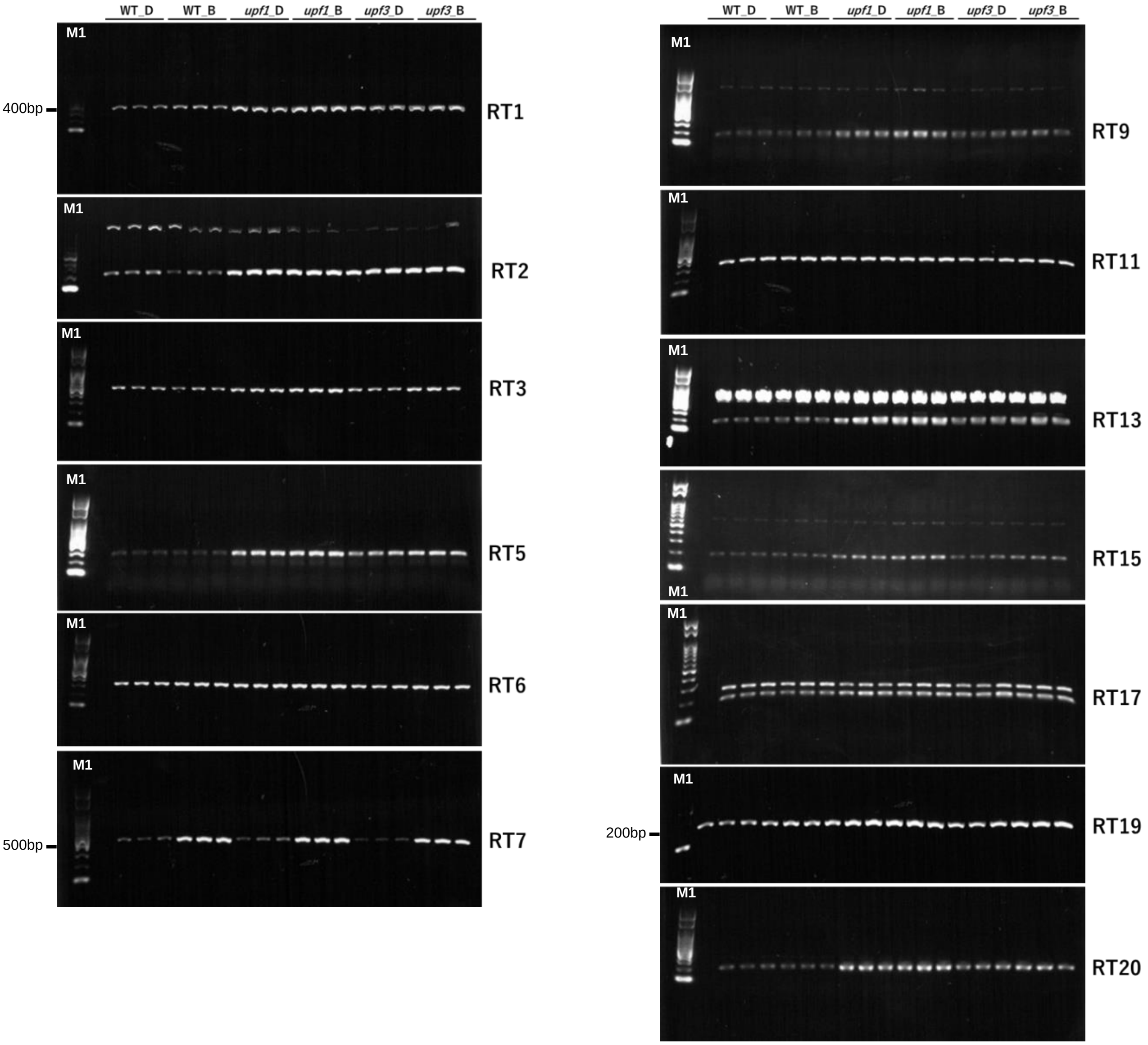

Unprocessed data for Fig. 2

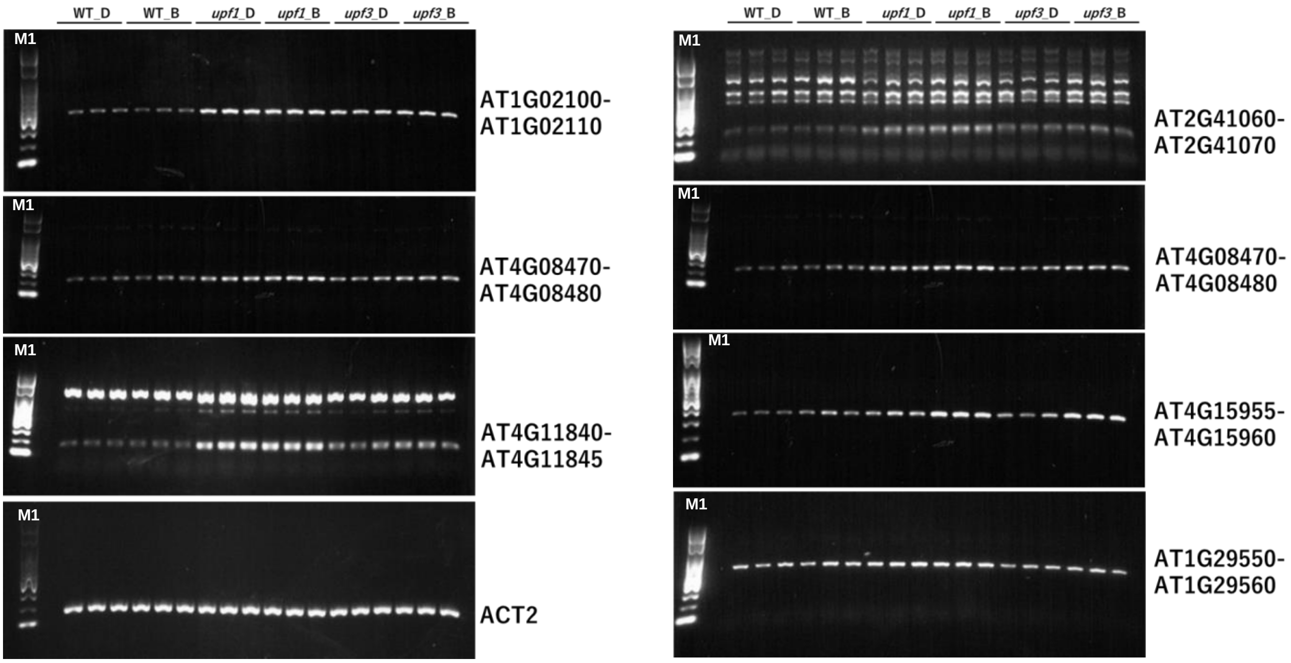

Unprocessed data for Fig. 3c

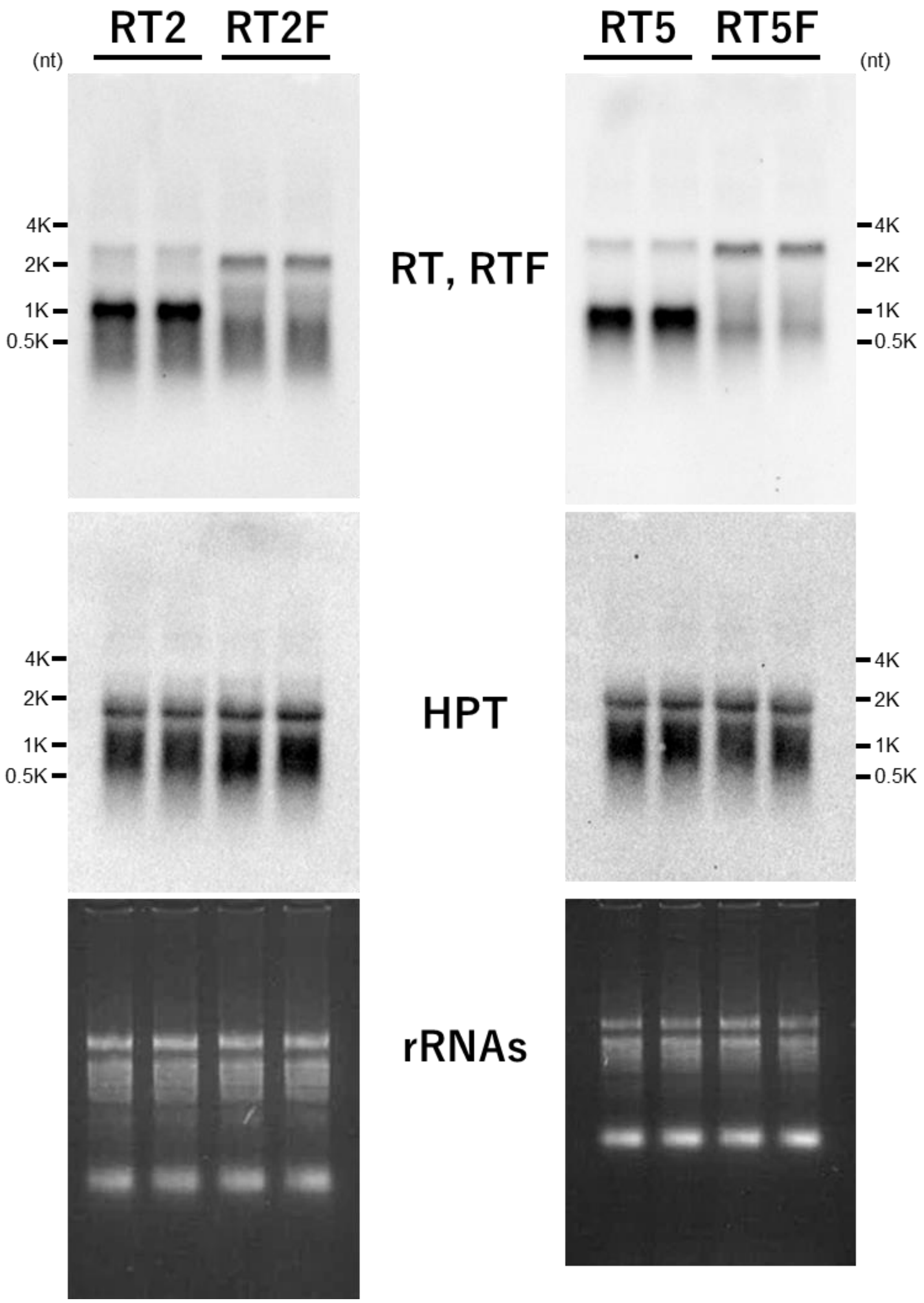

Unprocessed data for Fig. 3e

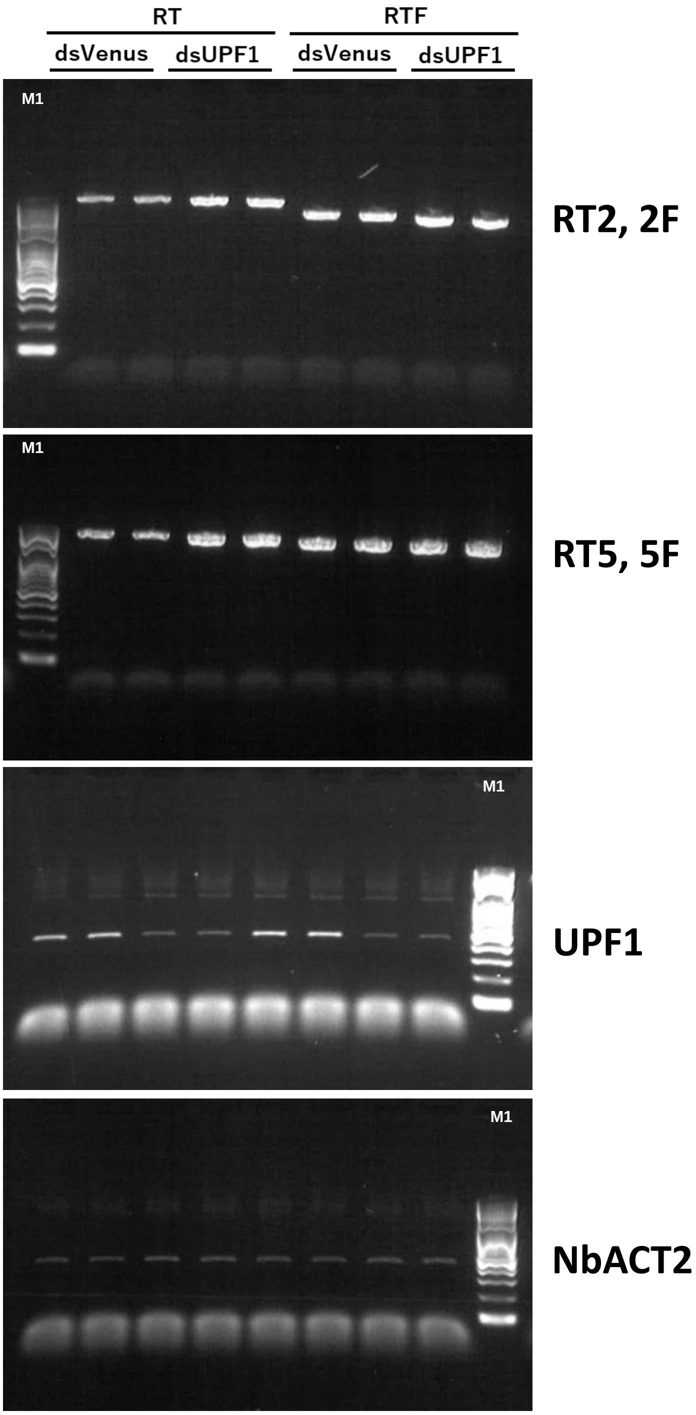

Unprocessed data for Fig. 4

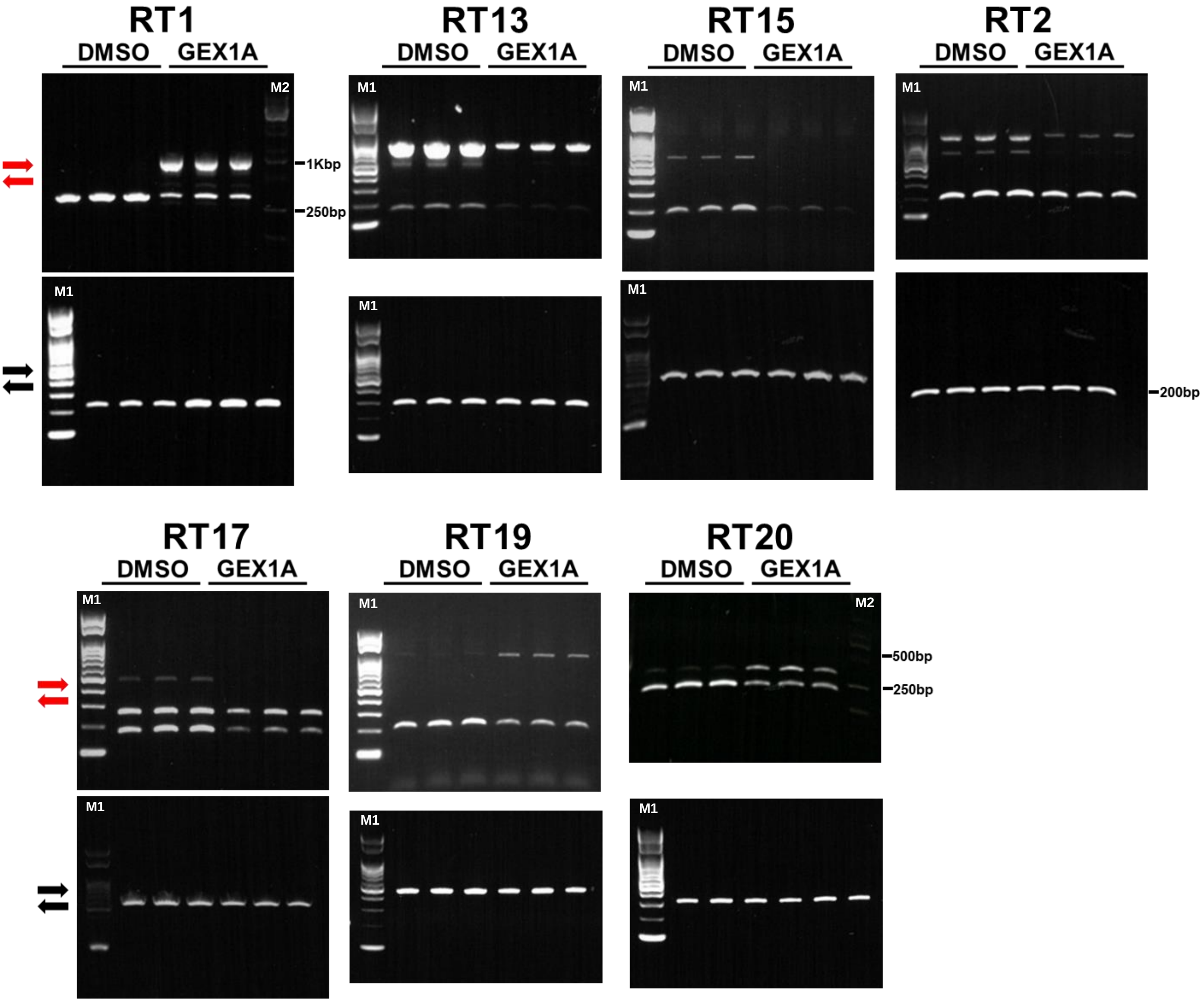

To know the bp length for the lower panel in RT2, the same DMSO sample was loaded with the M1 marker.

Unprocessed data for Fig. 5a

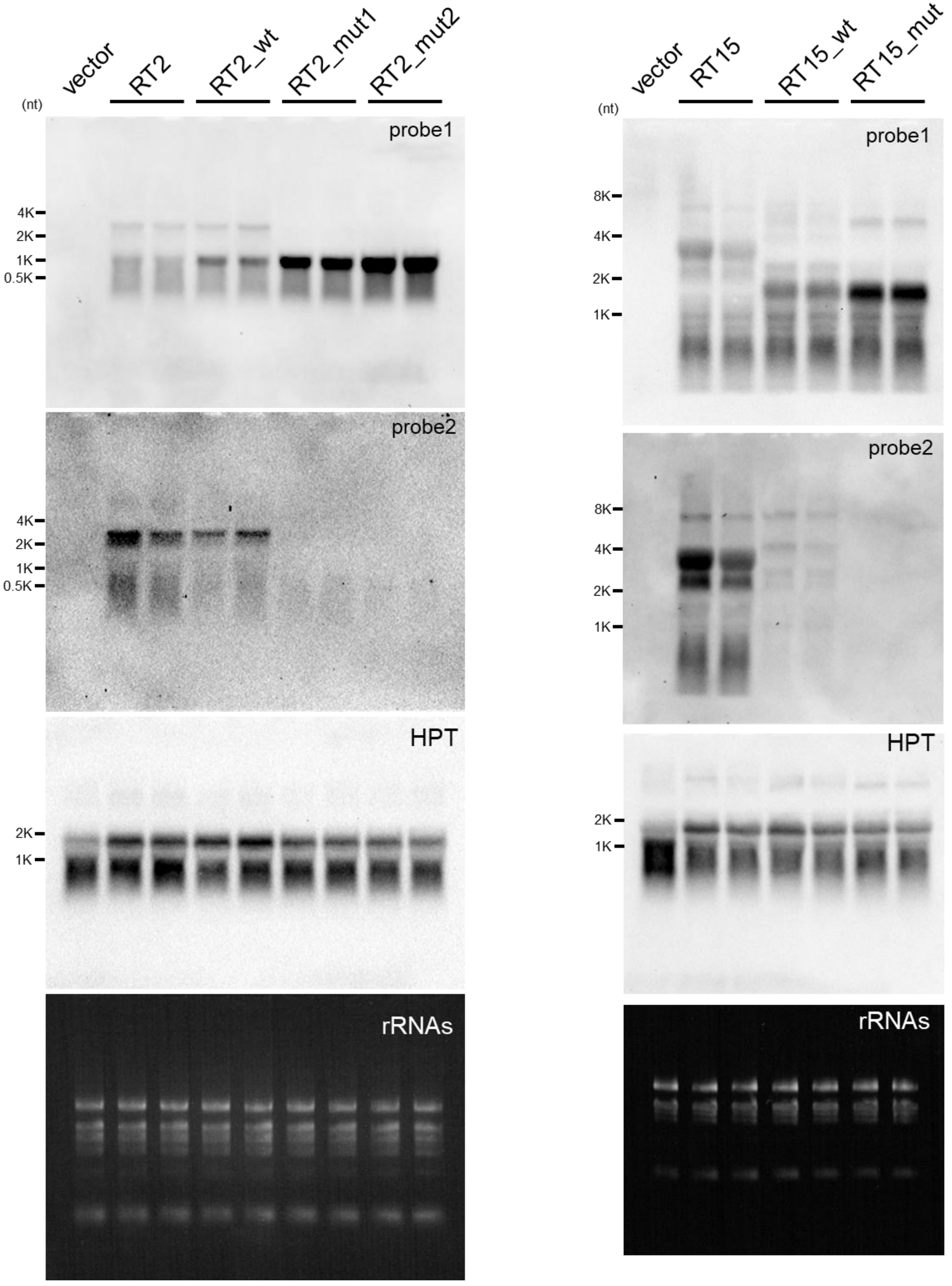

Unprocessed data for Fig. 5b

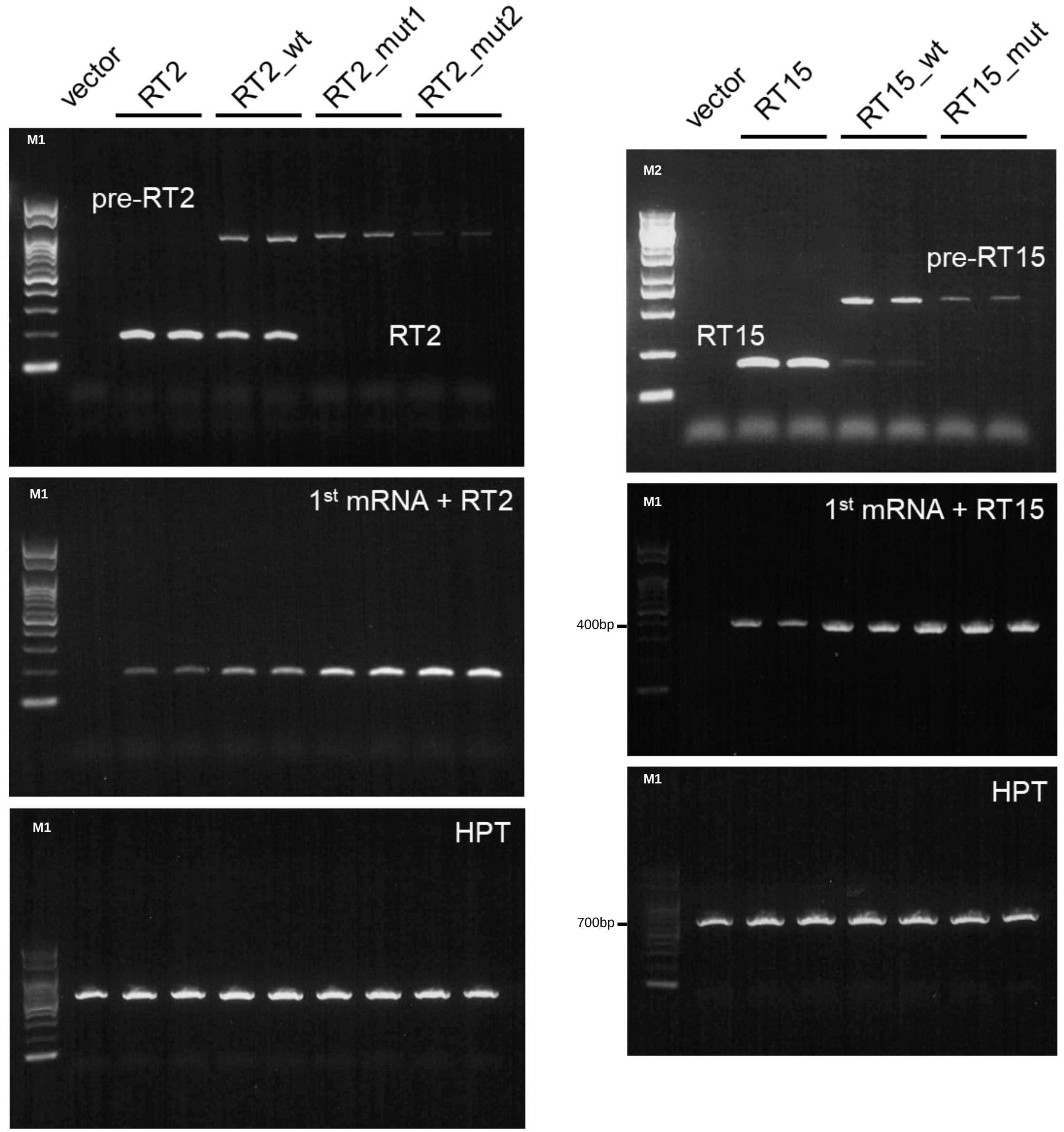

**Supplementary Table 1.** List of primers used for plasmid construction.	
Primer name	Sequence 5' to 3'
RT2_HiFi_F	TCATTTGGAGAGAACACGGGGGACT GATGCTCATCCATTAGAGGAGGA
RT2_HiFi_R	GAACGATCGGGGAAATTCGAGCTGC GAAAACCCAATGACAAACCAGA
RT1_HiFi_F	TCATTTGGAGAGAACACGGGGGACT GGTTTAGTTTCTGATCTGTTAAAG
RT1_HiFi_R	GAACGATCGGGGAAATTCGAGCTGC ATCAAAACCATATCAACGTTCA
RT3_HiFi_F	TCATTTGGAGAGAACACGGGGGACT ACACTCAAGAGGACGAGCCATTA
RT3_HiFi_R	GAACGATCGGGGAAATTCGAGCTGC CGAAGTTTCAATGTGATTGCAGT
RT5_HiFi_F	TCATTTGGAGAGAACACGGGGGACT ACCACTAAAGTTTAGGGACATA
RT5_HiFi_R	GAACGATCGGGGAAATTCGAGCTGC AGTACCAAACCGATCTGTATTG
RT6_HiFi_F	TCATTTGGAGAGAACACGGGGGACT AACCTAACCATGGAAATTGCA
RT6_HiFi_R	GAACGATCGGGGAAATTCGAGCTGC AGATGTATTTCTGTGATTTAC
RT7_HiFi_F	TCATTTGGAGAGAACACGGGGGACT TCCACCACAATCCCTCTCTC
RT7_HiFi_R	GAACGATCGGGGAAATTCGAGCTGC GAGGAAGCAGTAGTCTTTGA
RT9_HiFi_F	TCATTTGGAGAGAACACGGGGGACT ATAAGAAAAAACCAGAATTTTG
RT9_HiFi_R	GAACGATCGGGGAAATTCGAGCTGC GGTGCTGAAAAGAATGTATAC
RT11_HiFi_F	TCATTTGGAGAGAACACGGGGGACT CTGCTCTTCTTCCTCTTGTTG
RT11_HiFi_R	GAACGATCGGGGAAATTCGAGCTGC GGTAAATCTAAGGACATTGA
RT15_HiFi_F	TCATTTGGAGAGAACACGGGGGACT

	GAAGTTCTTCTTCCTCATCG
RT15_HiFi_R	GAACGATCGGGGAAATTCGAGCTGC AGACCGAAATTTCATTGACT
RT2_wt_IS_intron_F	GTCTGATTCGGGTTATGAACACAAG GTACCTTCTATTAAGCTTAGCAA
RT2_wt_IS_intron_R	TCCGTGACAGAGGTGACTGTGACAT CTGTAGAGAGAGAAGATAGGGTA
RT2_mut1_IS_intron_F	GTCTGATTCGGGTTATGAACACAAG CTCCCTTCTATTAAGCTTAGCAA
RT2_mut1_IS_intron_R	TCCGTGACAGAGGTGACTGTGACAT CCCTAGAGAGAGAAGATAGGGTA
RT2_5'exon_R	CTTGTGTTCATAACCCGAATCAGAC
RT2_3'exon_F	ATGTCACAGTCACCTCTGTCACGGA
RT2_mut2_minor_IS_intron _F	TTCGAGATTTTTGATCTACGTTAAG CTCACGACTTCTGTGGACTTATC
RT2_mut2_minor_IS_intron _R	GCCTTCTCAGACCAAGTATACAGTT CCCGTTAGGGTGGTGGTTATGAT
RT2_5'exon2_R	CTTAACGTAGATCAAAAATCTCGAA
RT2_3'exon2_F	AACTGTATACTTGGTCTGAGAAGGC
RT15_wt_IS_intron_F	TATATGGAAGGCACTAAATCTTCAG GTACTATTATTTAGTTTTCGG
RT15_wt_IS_intron_R	GTCAAAAGATATTTGACCCGCCCGT CTTGGACAGATTGGATTAAA
RT15_mut1_IS_intron_F	TATATGGAAGGCACTAAATCTTCAG CTCCTATTATTTAGTTTTCGG
RT15_mut1_IS_intron_R	GTCAAAAGATATTTGACCCGCCCGT CCCGGACAGATTGGATTAAA
RT15_5'exon_R	CTGAAGATTTAGTGCCTTCCATATA
RT15_3'exon_F	ACGGGCGGGTCAAATATCTTTTG
RT2_1st_R	ACGTAGATCAAAAATCTCGAACC
RT2_2nd_F	TAGGTTCGAGATTTTTGATCTACGT ATGATGATGATGAGAGGTGGT
RT5_1st_R	ATTAGGAATTTCAGGGATTGCATTG
RT5_2nd_F	CAATGCAATCCCTGAAATTCCTAAT ATGGCGGAACTAAAGCTATC

dsUPF1_F	CACCTCATTTCTGCTAAGGCAGAGAGTG
dsUPF1_R	AGACGCAACTCATTATCTTCCTTAG

**Supplementary Table 2.** List of primers used for RT-PCR (Figs. 2 and 5).	
Primer_name	Sequence 5' to 3'
AT2G41060-AT2G41070_F	GTCCTTACATGGGTCGTTAGATTAG
AT2G41060-AT2G41070_R	AGGCTCTTCAATGTTTCCTCTAATAG
AT2G01390-AT2G01400_F	TCTTGTTGCTTCTGCACGTTTCCGAG
AT2G01390-AT2G01400_R	GAAAGTGAGATCAAAACAGGTGTCCA
AT4G11840-AT4G11845_F	GGGACTTAGCTTTCCGATCCAAAGA
AT4G11840-AT4G11845_R	CAAACGGCACGAGTTGCTTGTCAAG
AT4G08470-AT4G08480_F	GTTTATGAGATATTAGACCCCATAGA
AT4G08470-AT4G08480_R	TTAGGGATAAGATACTCTCTGGATTG
AT4G25280-AT4G25290_F	TTCGTAAGCCTGAATCCGTAATGTC
AT4G25280-AT4G25290_R	TCAGAATCGAGCAAGAAATATCGTC
AT1G02100-AT1G02110_F	GATGATTGATTGCACAAAGTTAGAAAG
AT1G02100-AT1G02110_R	ACAGCATCCTCACTGTCGAGCTT
AT4G15955-AT4G15960_F	TCAACACATTCACAACTTCTTTCAC
AT4G15955-AT4G15960_R	GCTTTTCTCGGCGACGTGCATA
AT4G19880-AT4G19890_F	TGTCGCCTGTTCAGTAGATTCTAAAG
AT4G19880-AT4G19890_R	GTTGCTAATGATCGTGGAATAAGACA
AT3G26539-	TGATCATTACCTTCAGCCACCATGAATG

AT3G26540_F	
AT3G26539-AT3G26540_R	CAATGTCCATGGAGGCCACGATATAAG
AT1G29550-AT1G29560_F	CGTCGATATACTGTATGAGATTCAAC
AT1G29550-AT1G29560_R	CTCCAGCGTCTGCCATTAAAGTTGAGA
AT4G00030-AT4G00040_F	GGACTGAATCATTTGTATAATTTGTGATGC
AT4G00030-AT4G00040_R	ACCAACATCTTTGTGAGTTCACAATG
AT5G67630-AT5G67640_F	CTAATTAGGGTTCAAATTATCACAGAC
AT5G67630-AT5G67640_R	CATATTCGATTATCGTCTTCGTGCGA
AT4G03250-AT4G03260_F	GACGATGAGACTGACGAATCTAGC
AT4G03250-AT4G03260_R	ACTTTGCAGAAGCGGCCAGAGTGA
AT5G24610-AT5G24620_F	GTTACTCCTTAACTGTGAGAAGAAG
AT5G24610-AT5G24620_R	AGACTGTCCCACATCAAGCCTGAAC
AT1G10600-AT1G10610_F	CTGTATACTTGGTCTGAGAAGGCTG
AT1G10600-AT1G10610_R	ATATACGATCGTACAAGACAATGGTA
AT5G57140-AT5G57150_F	GGAAACTTCCGACTCATTTCTGAAG
AT5G57140-AT5G57150_R	ACTGTCTCTAGTTCGTTTCGTTGGTC
AT3G42630-AT3G42640_F	CTAGCTACATGCCACATCTTTGCTG
AT3G42630-AT3G42640_R	TCATCCCACGAGAATTCAGTCGCCA

AT3G50685-AT3G50690_F	CAAAGGAAGAGTTTCGAGACCTTG
AT3G50685-AT3G50690_R	CTCTCACCAGTTAATTACCACACA
AT1G78800-AT1G78810_F	GCTTAATCATATTGTTTCTCTTCTACG
AT1G78800-AT1G78810_R	GACTTGACTGGTTTACGTTCTTCTG
AT4G03250-AT4G03260_1st_F	ATCAAAGTTGCATTCGCCAGATC
AT4G03250-AT4G03260_1st_R	CATGAGACTGATTGGTTTCAATATTG
AT3G42630-AT3G42640_1st_F	GACGATTCTCCTCTTGTCTTAACTG
AT3G42630-AT3G42640_1st_R	CAAGCATCGGAAAAGTTTATCAGG
AT2G01390-AT2G01400_1st_F	GTGTAGCTGCTTACACTGCGCTGA
AT2G01390-AT2G01400_1st_R	ACCAGATTACAACAGATTTGTAGC
AT4G11840-AT4G11845_1st_F	CTCTCGTTTCAAATGTGGACAACC
AT4G11840-AT4G11845_1st_R	CTGAGTGTAGCAATCGATTGCGGT
AT4G08470-AT4G08480_1st_F	GTTGAAGCCCTGTTTAGGATCAGA
AT4G08470-AT4G08480_1st_R	TAGATTCATCTACGGATTAGCGGA
AT3G26539-AT3G26540_1st_F	ATGGATGCTTGCATCAGCTTGAAG
AT3G26539-AT3G26540_1st_R	AGCTCCGATATGACTGCAGGAGA
AT1G10600-AT1G10610_1st_F	TACGGGATATTTAAGCTAACGGAC
AT1G10600-AT1G10610_1st_R	CTTAACGTAGATCAAAAATCTCG

AT5G57140-AT5G57150_1st_F	AGCCAGAGGATTCATGTTAATCC
AT5G57140-AT5G57150_1st_R	AGTTTCCCATAAGACTTGTTCGTC
AT4G00030-AT4G00040_1st_F	ATTCAATAGTGCGGTATTGAGAGG
AT4G00030-AT4G00040_1st_R	CAAATGATTCAGTCCAGTTATAAG
AT5G24610-AT5G24620_1st_F	TGGAGACGGAATGGAGTTGGTTTAG
AT5G24610-AT5G24620_1st_R	CATGTTTCGTTGATAGTTTTGAAC
AT4G19880-AT4G19890_1st_F	CAACCTTGTTACTGATTTGACTG
AT4G19880-AT4G19890_1st_R	CACTCTCCAGTCTTTAGAATCTAC
AT5G67630-AT5G67640_1st_F	GACTAATCGGTGACAATGATAAGAC
AT5G67630-AT5G67640_1st_R	TGAACCCTAATTAGGAATTTCAGG
AT4G25280-AT4G25290_1st_F	CACGATGAAATCTCAGAAGAGG
AT4G25280-AT4G25290_1st_R	ACTGCTTTCTGATGACCAAATC
AT3G42630-AT3G42640_1st_F	GAGTTTGTATACAACCGAATGAATC
AT3G42630-AT3G42640_1st_R	ATTGTGATAGTGAGCAAAGAGTGAC
AT3G50685-AT3G50690_1st_F	GATGAGTAATGGCCATAGTGAAAG
AT3G50685-AT3G50690_1st_R	ATCATCATCTTCATCCGCAAACTC
AT1G78800-AT1G78810_1st_F	CAACAGGTCATGTCGGTTGAAGA
AT1G78800-AT1G78810_1st_R	AGCCAAGTAGTTCACAGACAAAC

HPT_F (HPT_Rprobe_F)	ATGAAAAAGCCTGAACTCAC
HPT_R (HPT_Rprobe_R)	ATAATACGACTCACTATAGGGTCCATCACAGTTTGCCAGTG

**Supplementary Table 3.** List of primers used for probe construction.	
Primer_name	Sequence 5' to 3'
2-1Rprobe_F	CACGTTTCTCGGGTTCTAATC
2-1Rprobe_R	ATAATACGACTCACTATAGGG TGGCACCATTACCTGATACG
2-2Rprobe_F	GATTCATGGAGAAGTAGTGATG
2-2Rprobe_R	ATAATACGACTCACTATAGGG ATCCTTTTCCTTGTTCATTATC
15-1Rprobe_F	ATGATGAATATTTACGACGATG
15-1Rprobe_R	ATAATACGACTCACTATAGGG CGACTGAGCTATCTTCTTCAC
15-2Rprobe_F	GAGAGATTGATTGTTGATGCG
15-2Rprobe_R	ATAATACGACTCACTATAGGG ATGTATTGGCAAATGTTGATG
1-1Rprobe_F	GCGTGTTATCGGCACCGGAAC
1-1Rprobe_R	ATAATACGACTCACTATAGGG CAGCACAATGCAGCTGCCTC
3-1Rprobe_F	CCTAAGTCGAATCGATTCCTTC
3-1Rprobe_R	ATAATACGACTCACTATAGGG TCAATCTCCCAATCTCCGAG
5-1Rprobe_F	AAGCGTCGAGAATTTCACGAAG
5-1Rprobe_R	ATAATACGACTCACTATAGGG TCATTGTCACCGATTAGTCC
6-1Rprobe_F	CTCTCGCTGTCGGCATGTTC
6-1Rprobe_R	ATAATACGACTCACTATAGGG CAGTTCTTCCCTCTCAATAC
7-1Rprobe_F	TCTCCCGCATTTCCTGCATC
7-1Rprobe_R	ATAATACGACTCACTATAGGG GAGTACGCCAAGCAACAATC
9-1Rprobe_F	TCAGATTCAGGCGAAGCGAC
9-2Rprobe_R	ATAATACGACTCACTATAGGG TCTCTTTACGGCAGTGATA
11-1Rprobe_F	ATGGAGGTATTTGGAAAATCTC
11-1Rprobe_R	ATAATACGACTCACTATAGGG ACCAACTCCATTCCGTCTCC
HPT_Rprobe_F	ATGAAAAAGCCTGAACTCAC
HPT_Rprobe_R	ATAATACGACTCACTATAGGGTCCATCACAGTTTGCCAGTG

**Supplementary Table 4.** List of primers used for RT-PCR of RNAi assay.	
Primer_name	Sequence 5' to 3'
NbUPF1_F	ATTAACTGCTCAAGAGGAGGTTG
NbUPF1_R	CTAATTGGTCCACAGCAACATTAC
NbACT_F	ACTGATGAAGATACTCACAGAAAG
NbACT_R	GAGCTAATGCAGTAATTTCCTTG
AT1G10600-AT1G10610_Long_F	TACGGGATATTTAAGCTAACGGAC
AT1G10600-AT1G10610_Long_R	GAAAACCCAATGACAAACCAG
AT5G67630-AT5G67640_Long_F	GACTAATCGGTGACAATGATAAGAC
AT5G67630-AT5G67640_Long_R	CATAAATCCGAGTTCAACATC
