# Supplementary material for: Intergenic splicing-stimulated transcriptional readthrough is suppressed by nonsense-mediated mRNA decay in Arabidopsis
Source: Commun Biol. 2022 Dec 20;5:1390. doi: 10.1038/s42003-022-04348-y (PMC9768141; doi:10.1038/s42003-022-04348-y)
Supplement: Supplementary file 2 — Description of Additional Supplementary Files [file 42003_2022_4348_MOESM2_ESM.pdf]

Description of Additional Supplementary Files

File name: Supplementary Data 1
Description: List of RT loci identified in ISOseq analysis.

File name: Supplementary Data 2
Description: Source data for Figs. 3B and 3D.

File name: Supplementary Data 3
Description: Addgene IDs for plasmids.
