# Supplementary material for: Intergenic splicing-stimulated transcriptional readthrough is suppressed by nonsense-mediated mRNA decay in Arabidopsis
Source: Commun Biol. 2022 Dec 20;5:1390. doi: 10.1038/s42003-022-04348-y (PMC9768141; doi:10.1038/s42003-022-04348-y)
Supplement: Supplementary file 6 — Reporting Summary [file 42003_2022_4348_MOESM6_ESM.pdf]

nature portfolio

Corresponding author(s): Minami Matsui

Last updated by author(s): Nov 22, 2022

Reporting Summary

Nature Portfolio wishes to improve the reproducibility of the work that we publish. This form provides structure for consistency and transparency in reporting. For further information on Nature Portfolio policies, see our Editorial Policies and the Editorial Policy Checklist.

Statistics

For all statistical analyses, confirm that the following items are present in the figure legend, table legend, main text, or Methods section.

n/a	Confirmed	
☐	☒	The exact sample size (*n*) for each experimental group/condition, given as a discrete number and unit of measurement
☐	☒	A statement on whether measurements were taken from distinct samples or whether the same sample was measured repeatedly
☐	☒	The statistical test(s) used AND whether they are one- or two-sided<br>*Only common tests should be described solely by name; describe more complex techniques in the Methods section.*
☐	☒	A description of all covariates tested
☐	☒	A description of any assumptions or corrections, such as tests of normality and adjustment for multiple comparisons
☐	☒	A full description of the statistical parameters including central tendency (e.g. means) or other basic estimates (e.g. regression coefficient) AND variation (e.g. standard deviation) or associated estimates of uncertainty (e.g. confidence intervals)
☐	☒	For null hypothesis testing, the test statistic (e.g. *F*, *t*, *r*) with confidence intervals, effect sizes, degrees of freedom and *P* value noted<br>*Give P values as exact values whenever suitable.*
☒	☐	For Bayesian analysis, information on the choice of priors and Markov chain Monte Carlo settings
☒	☐	For hierarchical and complex designs, identification of the appropriate level for tests and full reporting of outcomes
☒	☐	Estimates of effect sizes (e.g. Cohen's *d*, Pearson's *r*), indicating how they were calculated

Our web collection on statistics for biologists contains articles on many of the points above.

Software and code

Policy information about availability of computer code

Data collection

Publicly available data were downloaded and no specific software was applied.

Data analysis

The following softwares were used in our data processing:
- Ribo-seq/RNA-seq
FASTX Toolkit 0.0.14, fastq_illumina_filter (version 0.1), Bowtie version 2.3.4.1, TopHat version 2.1.1, RiboSeq package version 20170402 (https://github.com/ingolia-lab/RiboSeq), R version 4.0.2, R library of DESeq 1.42.0
STAR version 020201, Cufflinks v2.2.1

- CAGE
BWA v0.7.12, HISAT2 v2.0.5, CAGEr software v1.16.0

- ISO-seq
In the SMRT Link ver. 9.0.0 (PacBio), lima 1.11.0 (commit v1.11.0-1-gec618c9), isoseq3 3.3.0 (commit v3.3.0-1-gddba0c8) and pbmm2 1.2.0 (commit v1.2.0-1-g31b4be0) were used.

- Nanopore
EPI2ME Desktop Agent v3.4.2, minimap2 version 2.20-r1064-dirty

- Common
samtools version 1.6, spliced_bam2gff version 1.2

- Others
BUSCO v5.1.3

For manuscripts utilizing custom algorithms or software that are central to the research but not yet described in published literature, software must be made available to editors and reviewers. We strongly encourage code deposition in a community repository (e.g. GitHub). See the Nature Portfolio guidelines for submitting code & software for further information.

Data

Policy information about availability of data

All manuscripts must include a data availability statement. This statement should provide the following information, where applicable:

- Accession codes, unique identifiers, or web links for publicly available datasets
- A description of any restrictions on data availability
- For clinical datasets or third party data, please ensure that the statement adheres to our policy

The data set of sequenced reads by the next-generation sequencers is deposited in the DDBJ/EMBL/GenBank BioProject under accession number of DRA014187 and DRR Run number of DRR377382-DRR377434. Source data of Figs. 3b and 3d are provided in Supplementary Data 2. The Addgene IDs of newly-constructed plasmids were available in Supplementary Data 3. Uncropped data are shown at the end of Supplementary Information.

Human research participants

Policy information about studies involving human research participants and Sex and Gender in Research.

Reporting on sex and gender	*Use the terms sex (biological attribute) and gender (shaped by social and cultural circumstances) carefully in order to avoid confusing both terms. Indicate if findings apply to only one sex or gender; describe whether sex and gender were considered in study design whether sex and/or gender was determined based on self-reporting or assigned and methods used. Provide in the source data disaggregated sex and gender data where this information has been collected, and consent has been obtained for sharing of individual-level data; provide overall numbers in this Reporting Summary. Please state if this information has not been collected. Report sex- and gender-based analyses where performed, justify reasons for lack of sex- and gender-based analysis.*
Population characteristics	*Describe the covariate-relevant population characteristics of the human research participants (e.g. age, genotypic information, past and current diagnosis and treatment categories). If you filled out the behavioural & social sciences study design questions and have nothing to add here, write "See above."*
Recruitment	*Describe how participants were recruited. Outline any potential self-selection bias or other biases that may be present and how these are likely to impact results.*
Ethics oversight	*Identify the organization(s) that approved the study protocol.*

Note that full information on the approval of the study protocol must also be provided in the manuscript.

Field-specific reporting

Please select the one below that is the best fit for your research. If you are not sure, read the appropriate sections before making your selection.

☒ Life sciences ☐ Behavioural & social sciences ☐ Ecological, evolutionary & environmental sciences

For a reference copy of the document with all sections, see nature.com/documents/nr-reporting-summary-flat.pdf

Life sciences study design

All studies must disclose on these points even when the disclosure is negative.

Sample size	Three-day-old Arabidopsis thaliana wild-type (WT) and upf1-1 seedlings grown in the dark or blue-light irradiated following growth in darkness were used for Iso-seq, Nanopore sequencing, CAGE, RNA-seq, Ribo-seq and quantitative RT-PCR analyses. Leaves of one-month-old Nicotiana benthamiana were used for transient expression assay for Ribo-seq, RNA-seq, quantitative RT-PCR and Northern blot analysis.
Data exclusions	No data were excluded
Replication	Each one replicate of Iso-seq and Nanopore sequencing was performed, where Nanopore sequencing was used instead of multiple replicates of Iso-seq. Three replicates of Arabidopsis CAGE and RNA-seq were performed, where both libraries were constructed from the same samples. Two replicates of Arabidopsis Ribo-seq and RNA-seq were performed, where both libraries were constructed from the same samples. Three replicates of Nicotiana benthamiana Ribo-seq and RNA-seq were performed, where both libraries were constructed from the same samples. Three replicates of quantitative RT-PCR were performed on Arabidopsis samples and two replicates of quantitative RT-PCR and Northern blot were performed on Nicotiana benthamiana samples.

Randomization	The samples in each group were randomly selected and separately measured.
Blinding	The investigators were blinded to group allocation during data collection and analysis.

Reporting for specific materials, systems and methods

We require information from authors about some types of materials, experimental systems and methods used in many studies. Here, indicate whether each material, system or method listed is relevant to your study. If you are not sure if a list item applies to your research, read the appropriate section before selecting a response.

Materials & experimental systems

n/a	Involved in the study
☒	☐ Antibodies
☒	☐ Eukaryotic cell lines
☒	☐ Palaeontology and archaeology
☒	☐ Animals and other organisms
☒	☐ Clinical data
☒	☐ Dual use research of concern

Methods

n/a	Involved in the study
☒	☐ ChIP-seq
☒	☐ Flow cytometry
☒	☐ MRI-based neuroimaging
